# Closed-loop recruitment of striatal interneurons prevents compulsive-like grooming behaviors

**DOI:** 10.1038/s41593-024-01633-3

**Published:** 2024-05-01

**Authors:** Sirenia Lizbeth Mondragón-González, Christiane Schreiweis, Eric Burguière

**Affiliations:** Institut du Cerveau - Paris Brain Institute - ICM, Sorbonne Université, Inserm, CNRS, AP-HP Hôpital de la Pitié Salpêtrière, Paris, France

**Keywords:** Neural circuits, Psychiatric disorders, Predictive markers

## Abstract

Compulsive behaviors have been associated with striatal hyperactivity. Parvalbumin-positive striatal interneurons (PVIs) in the striatum play a crucial role in regulating striatal activity and suppressing prepotent inappropriate actions. To investigate the potential role of striatal PVIs in regulating compulsive behaviors, we assessed excessive self-grooming—a behavioral metric of compulsive-like behavior—in male *Sapap3* knockout mice (*Sapap3*-KO). Continuous optogenetic activation of PVIs in striatal areas receiving input from the lateral orbitofrontal cortex reduced self-grooming events in *Sapap3*-KO mice to wild-type levels. Aiming to shorten the critical time window for PVI recruitment, we then provided real-time closed-loop optogenetic stimulation of striatal PVIs, using a transient power increase in the 1–4 Hz frequency band in the orbitofrontal cortex as a predictive biomarker of grooming onsets. Targeted closed-loop stimulation at grooming onsets was as effective as continuous stimulation in reducing grooming events but required 87% less stimulation time, paving the way for adaptive stimulation therapeutic protocols.

## Abstract (web summary)

This paper shows that compulsive-like grooming in *Sapap3-*knockout mice can be reduced by closed-loop optogenetic stimulation of striatal interneurons based on grooming onset prediction, suggesting that adaptive stimulation may have therapeutic potential in obsessive–compulsive disorder.

## Main

Compulsive behaviors consist of pathological repetitive behaviors executed despite negative consequences. They are a core feature of various neuropsychiatric disorders, including obsessive–compulsive disorder (OCD). Increasing evidence in studies of neuropsychiatric disorders with repetitive behaviors points toward malfunctioning corticobasal ganglia circuits, which are key players in the formation and regulation of actions^1,2^. In particular, altered frontostriatal circuits including the orbitofrontal cortex (OFC) and its primary striatal input site have been observed in OCD patients^3,4^, as well as in rodent models expressing pathological repetitive behaviors, including, in particular, pathologically frequent self-grooming^5–7^. Electrical deep brain stimulation (DBS) of these striatal areas has proven efficient in reducing symptoms of OCD patients^8^. Striatal hyperactivity has surfaced as one of the prominent physiological characteristics during compulsive episodes^6,7,9^, and the reduction of striatal activity correlates with symptom alleviation in OCD patients^10,11^. However, the neuronal abnormalities that underlie this striatal hyperactivity in compulsive behaviors remain to be characterized. Under normal conditions, fast-spiking PVIs receive strong afferents from the cortex and regulate the activity of striatal medium spiny neurons (MSN) through a powerful feedforward inhibition, given their earlier and low activation threshold relative to MSN^10,12–15^. These physiological properties have been proposed to adapt and regulate behavioral output via the orchestration and tuning of MSN activity^16–19^. Postmortem studies in patients and mouse models suffering from pathological repetitive behaviors have reported a consistent decrease in PVI density in medial striatal areas^20–23^. This previous evidence suggests a crucial link between decreased PVI function and pathological repetitive behaviors, but a causal link has been lacking to date. To study the contributions of PVI in regulating compulsive behaviors, we used the *Sapap3*-KO mouse model that displays compulsive-like self-grooming, striatal hyperactivity and a decreased density of PVI in medial striatal areas^6,24,25^. To prove and precisely define their role in the emergence of transient and spontaneous compulsive-like self-grooming behaviors, we have established a closed-loop (CL) optogenetic approach with a real-time response system triggered by the detection of a reliable, symptom-predictive biomarker.

## Results

### Striatal PVI control compulsive-like grooming behavior

**Fig. 1:**
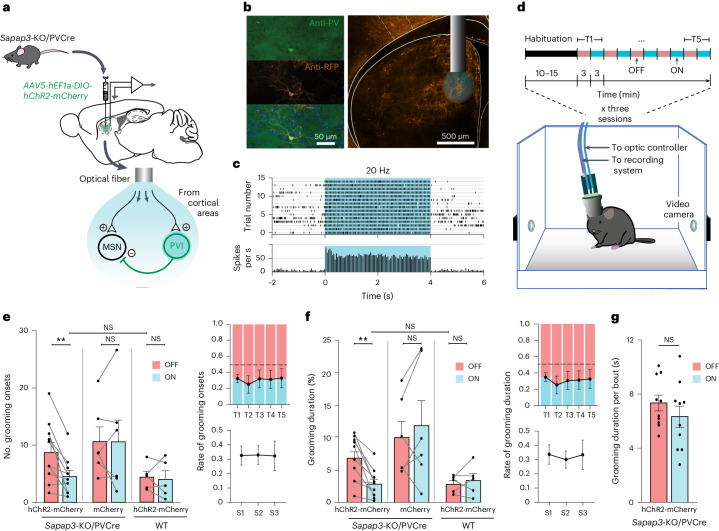
Optogenetic activation of striatal PVIs reduces compulsive-like self-grooming behavior in *Sapap3*-KO mice. **a**, Schematic illustration of the experimental design and simplified model of the bilateral optogenetic neuromodulation of PVI microcircuitry in the striatal areas receiving main lOFC input. **b**, Representative post hoc histological image illustrating striatal optic fiber placement and specific opsin expression in PVI. Fiber placement and opsin expression were verified in all animals, including *n* = 10 *Sapap3*-KO/PVCre mice and *n* = 5 WT mice. **c**, Spike raster plot (top) and peristimulus time histogram (bottom) confirming optogenetic recruitment of an hChR2-expressing PVI during the light stimulation period (cyan rectangles) over resting state. **d**, Experimental setup and optogenetic stimulation paradigm with alternating 3-min blocks of OFF (red bars) and ON (cyan bars) stimulation (20 Hz, 5 ms, 10 mW) trials (T). **e**,**f**, Optogenetic activation of PVI in the described striatal area reduces the number of grooming onsets (**e**) and the percentage of total experimental time spent grooming (**f**) in *Sapap3*-KO/PVCre mice expressing hChR2 (*n* = 10, Wilcoxon matched-pairs signed-rank, two-tailed tests, **P_ON–OFF, onsets_ = 0.004, **P_ON–OFF, duration_ = 0.002) to WT levels (*n* = 5) (Mann–Whitney, two-tailed tests, *P*_onsets_ = 0.93, *P*_duration_ = 0.78). The same injection and stimulation protocol did not alter grooming behavior either in *Sapap3*-KO/PVCre mice injected with a control virus, expressing only the fluorophore marker mCherry (*n* = 6, Wilcoxon matched-pairs signed-rank two-tailed test, *P*_ON–OFF, onsets_ = 1, *P*_ON–OFF, duration_ = 0.31), or in WT mice (*n* = 5, Wilcoxon matched-pairs signed-rank two-tailed tests, *P*_ON–OFF, onsets_ = 0.81, *P*_ON–OFF, duration_ = 0.81). Optogenetic treatment was effective across trials and sessions (right panels). **g**, Optogenetic stimulation did not affect the average duration of individual grooming events in *Sapap3*-KO/PVCre mice (*n* = 10, Wilcoxon matched-pairs signed-rank two-tailed tests, *P* = 0.21). Datapoints, single mice; bars, mean values ± s.e.m.; NS, nonsignificant. Source data

**Fig. 2:**
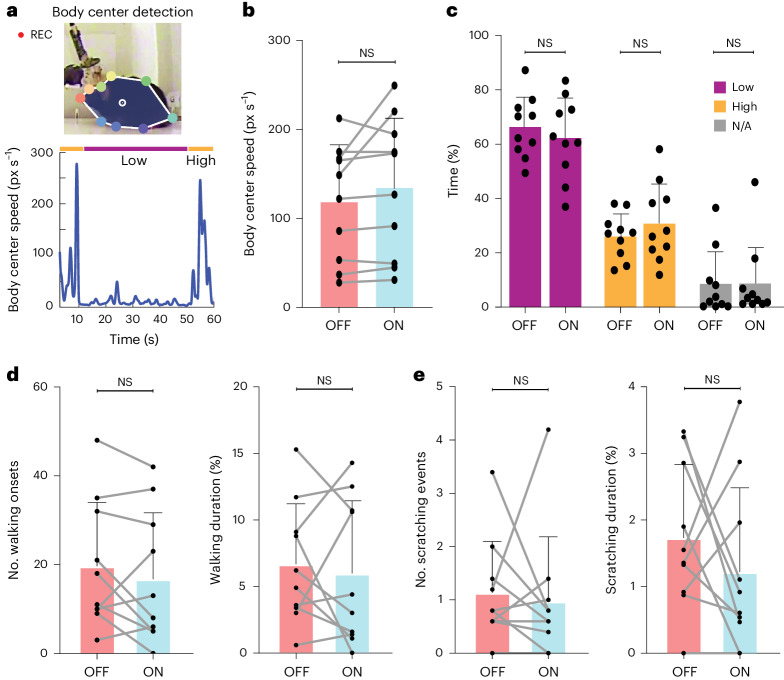
Optogenetic neuromodulation of striatal PVIs specifically decreases compulsive-like grooming rather than general activity in *Sapap3*-KO mice. **a**, We used video recordings to track specific body parts of the mice, calculate body center and measure overall activity. px, pixel. **b**, Global activity did not change significantly between OFF and ON stimulation periods (*M*_OFF_ = 118 ± 64.92, *M*_ON_ = 134.2 ± 78.61, *P*_OFF–ON_ = 0.1309). **c**, Likewise, no difference was detected when differentiating between low and high activity levels (*M*_OFF, Low_ = 65.85 ± 11.33, *M*_ON, Low_ = 61.81 ± 14.81, *P*_OFF–ON, Low_ = 0.5566; *M*_OFF, High_ = 25.87 ± 8.33, *M*_ON, High_ = 30.64 ± 14.42, *P*_OFF–ON, High_ = 0.1309). The small proportion of experimental time, in which the body center could not be tracked by video analysis, was comparable for both experimental conditions (*M*_OFF, N/A_ = 8.27 ± 12.12, *M*_ON, N/A_ = 7.55 ± 14.08, *P*_OFF–ON, NA_ = 0.6875). The video recording used had a frame rate of 25 fps and a frame size of 704 × 506 pixels. **d**, Neither the number (*M*_OFF_ = 19.7 ± 14.33, *M*_ON_ = 16.80 ± 14.91, *P*_ON–OFF, walking onsets_ = 0.27) nor the relative time spent walking (*M*_OFF_ = 6.66 ± 4.522, *M*_ON_ = 5.96 ± 5.441, *P*_ON–OFF, walking onsets_ = 0.7) was affected by optogenetic stimulation in *Sapap3*-KO mice. **e**, Optogenetic activation does not alter repetitive scratching (*M*_OFF_ = 1.14 ± 0.9617, *M*_ON_ = 0.98 ± 1.209, *P*_ON–OFF, scratching_ = 0.5781) or the percentage of time spent scratching (*M*_OFF_ = 1.737 ± 1.099, *M*_ON_ = 1.226 ± 1.26, *P*_ON–OFF, Scratching %_ = 0.4258). Statistical analyses were done using Wilcoxon matched-pairs signed-rank two-tailed tests. Each datapoint in **b**–**e** represents one of the *n* = 10 *Sapap3*-KO/PVCre mice analyzed across five independent 3-min trials. Bars, mean values ± s.d. Source data

**Extended Data Fig. 1:**
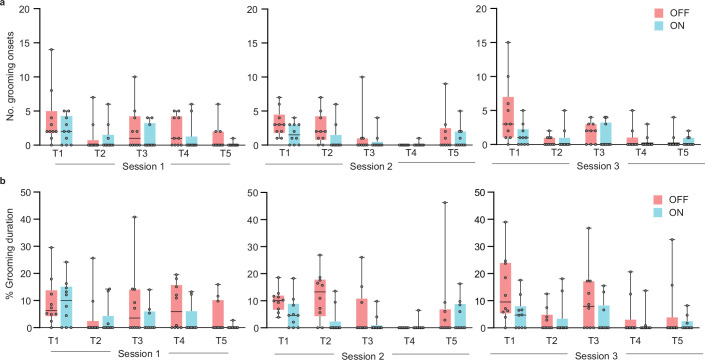
Detailed behavioural results of optogenetic stimulation of striatal PV interneurons in Sapap3-KO/PVCre mice in the striatal area receiving major lOFC projections. (**a**) Number of grooming onsets and (**b**) the percentage of grooming duration across individual consecutive trial blocks (T1-T5) and three separate sessions (1-3). Each graph depicts n = 10 Sapap3-KO/PV-Cre mice results, and the data are presented as mean values + SEM. Source data

**Extended Data Fig. 2:**
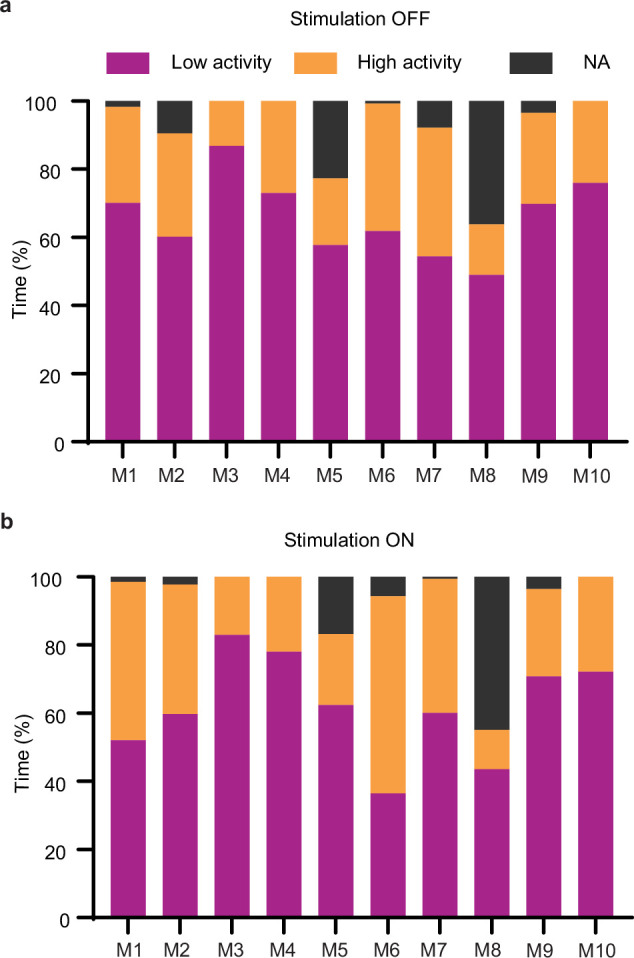
Individual distribution into low and high activity phases during OFF and ON stimulation conditions in Sapap3-KO/PVCre mice. Percentage of time individual mice spent in phases of low and high activity during periods (**a**) without optogenetic stimulation and (**b**) under optogenetic stimulation. The black bars correspond to uncertain periods of tracking due to insufficient recognition of body parts from video analysis. Each bar corresponds to an individual Sapap3-KO/PV-Cre mice (M1:M10). Source data

We injected *Sapap3*^−/−^::PV^Cre/Wt^ (*Sapap3*-KO/PVCre) mice (*n* = 10) bilaterally with a Cre-dependent adeno-associated viral vector expressing channelrhodopsin-2 (AAV5-hChR2(H134R)-mCherry) into the striatal areas receiving input from the lateral OFC (Fig. 1a,b)^26–30^. Bilateral implantation of optic fibers at the injection site allowed us to selectively recruit PVIs in the targeted striatal area (Fig. 1c and Supplementary Fig. 1) via a customized implant designed for simultaneous optogenetic neuromodulation and in vivo electrophysiology (Supplementary Fig. 2a–e). We videoed naïve freely moving *Sapap3*-KO/PVCre mice on three separate days while delivering optogenetic stimulation (5-ms pulses at 20 Hz, 10 mW). The experimental paradigm consisted of a 10- to 15-min habituation period, followed by ten alternating trials of 3 min of active blue-light stimulation (ON) and 3 min without blue-light stimulation (OFF; Fig. 1d). During periods of PVI optogenetic activation, *Sapap3*-KO/PVCre mice exhibited a significant 55.8% decrease in grooming bouts and 46.25% decrease in the experimental time spent grooming (Wilcoxon matched-pairs signed-rank tests, *P*_ON–OFF, onsets_ = 0.004, *P*_ON–OFF, duration_ = 0.002), reducing their grooming activity to levels comparable with those of wild-type (WT) baselines (Mann–Whitney test, *P*_onsets_ = 0.93, *P*_duration_ = 0.78) (Fig. 1e,f). This effect was consistent across trials (upper panels; Fig. 1e,f) and sessions (generalized linear mixed model (GLMM)—Type II Wald chi-square test: Chisq_onsets_ = 6.913, d.f._onsets_ = 1, *P*_onsets_ = 0.009; Chisq_ON–OFF duration_ = 18,848, d.f._ON–OFF duration_ = 1, *P*_ON–OFF, duration_ < 0.0001) (lower panels; Fig. 1e,f and Extended Data Fig. 1a,b). On the contrary, under the same protocol, age-matched control groups including *Sapap3*-KO/PVCre mice expressing a virus with a fluorescent reporter only (AAV5-mCherry) (*n* = 6) and WT (*Sapap3*^+/+^::PV^Cre/wt^) expressing hChR2-mCherry (*n* = 5) did not decrease their grooming activity significantly during the blue-light stimulation trials (Wilcoxon matched-pairs signed-rank test: *Sapap3*-KO/PVCre expressing mCherry: *P*_ON–OFF, onsets_ = 1, *P*_ON–OFF, duration_ = 0.31; WT mice expressing hChR2-mCherry: *P*_ON–OFF, onsets_ = 0.81, *P*_ON–OFF, duration_ = 0.81) (Fig. 1e,f). We looked at individual grooming bouts to further understand the reduction in grooming activity upon optogenetic excitation of striatal PVI in *Sapap3*-KO/PVCre expressing hChR2-mCherry. We found that the average duration of individual grooming events was consistent between ON and OFF trials (Fig. 1g; *n* = 10 animals, averaged across three sessions and five ON–OFF trials per session; Wilcoxon matched-pairs signed-rank tests, *P* = 0.21). Hence, the overall decrease in grooming duration (Fig. 1f) cannot be explained by a reduction in the duration of individual grooming events, but rather by a reduction in the number of grooming bout initiations. In contrast to the observed decrease in self-grooming behavior in *Sapap3*-KO/PVCre mice during ON stimulation periods, general activity (Wilcoxon matched-pairs signed-rank tests, *P*_ON–OFF_ = 0.13), phases of low and high activity (Wilcoxon matched-pairs signed-rank tests: *P*_ON–OFF, low_ = 0.56; *P*_ON–OFF, high_ = 0.13), walking behavior (Wilcoxon matched-pairs signed-rank tests: *P*_ON–OFF, number walking events_ = 0.27; *P*_ON–OFF, relative time spent walking_ = 0.7) and repetitive behaviors other than self-grooming remained unaltered (Wilcoxon matched-pairs signed-rank tests: *P*_ON–OFF, number scratching events_ = 0.58; *P*_ON–OFF, relative time spent scratching_ = 0.43) (Fig. 2a–e and Extended Data Fig. 2a,b). Our findings show that selective stimulation of striatal PVI activity alleviates excessive self-grooming whereas other behaviors remained unaltered. In particular, striatal PVI stimulation reduces the number of grooming onsets (GO), suggesting their implication in regulating grooming initiations.

### lOFC delta band activity predicts grooming events

**Fig. 3:**
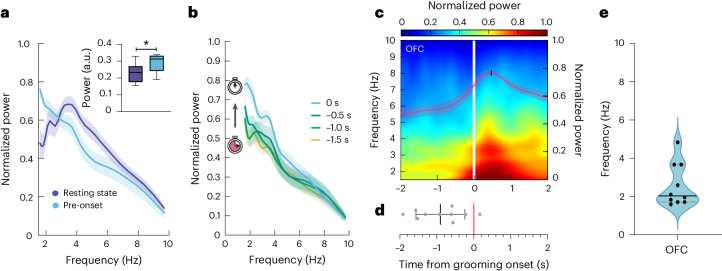
Low-frequency LFP signature in the OFC precedes grooming onset in *Sapap3*-KO mice. **a**, Average power spectral density of OFC LFPs recorded during 1-s periods before grooming onset and resting state in *Sapap3*-KO mice. The inset illustrates that average OFC LFP power in the 1.5–3 Hz range during pregrooming exceeds that of the resting state during the same period (*n* = 15 events for each condition per animal; Wilcoxon matched-pairs signed-rank two-tailed test, *P = 0.01). The box plots show medians (*M*_resting_ = 0.23, *M*_pre-onset_ = 0.31) and 25th (Q1_resting_ = 0.18, Q1_pre-onset_ = 0.24) and 75th (Q3_resting_ = 0.27, Q3_pre-onset_ = 0.33) percentiles, with whiskers at minimum (Min_resting_ = 0.15, Min_pre-onset_ = 0.19) and maximum (Max_resting_ = 0.33, Max_pre-onset_ = 0.34) values. **b**, Timecourse of average OFC power spectral density in incrementing 500-ms time windows before grooming onset of *Sapap3*-KO mice. **c**, The average spectrogram for continuous wavelet transform of OFC activity in *Sapap3*-KO mice displayed a progressive low-frequency power increase before grooming onset (vertical white line). The superimposed red trace represents the normalized power of the average 1.5–4 Hz frequency band (right-hand *y* axis). **d**, Power rising points of the 1.5–4 Hz time–frequency curve are observed on average 1 s before grooming onset (*M* = −0.9056, s.d. = 0.66). **e**, Frequency values at maximal power are centered on average around 2 Hz within 4-s perigrooming onset. The violin plot shows the median (*M* = 2.04), 25th (Q1 = 1.71) and 75th (Q3 = 3.67) percentile values. Envelopes in **a**–**c** represent ± s.e.m.; data in **d** represent mean ± s.d. and data in **e** represent median and interquartile range. Each graph depicts the results of *n* = 10 *Sapap3*-KO mice. Source data

**Fig. 4:**
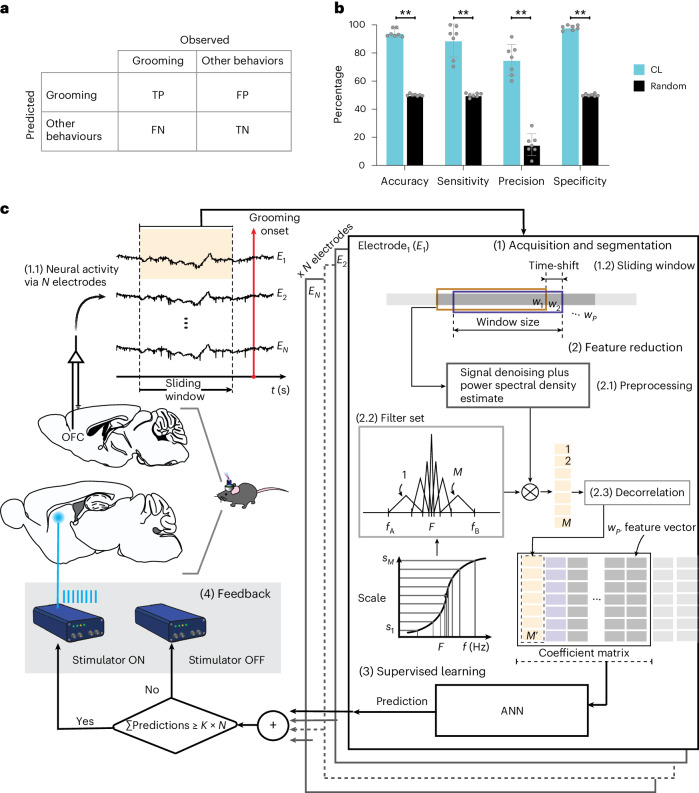
Machine-learning-based OFC LFP decoding predicts grooming onset in real time. **a**, Confusion matrix to assess the algorithm’s predictions with TP (correct prediction of grooming), TN (correct prediction of behaviors other than grooming), FP (other behaviors incorrectly predicted as grooming) and FN (grooming events falsely predicted as other behaviors). **b**, The metrics in **a** were used to calculate the average classification performance of the algorithm, including accuracy, sensitivity, precision and specificity. This evaluation was performed in an experimental design on continuous 10-min recordings from *n* = 7 *Sapap3*-KO/PVCre mice (average of 12.9 ± 5.1 grooming bouts per recording) to distinguish between TP and FP classifications. The results were compared with a uniform pseudorandom distribution; the results were found to be superior in terms of accuracy (94.2 ± 2.8 versus 50.1 ± 0.7, **P = 0.0078), sensitivity (88.8 ± 11.8 versus 50.1 ± 1.4, **P = 0.0078), precision (74.9 ± 11.2 versus 14.8 ± 7, **P = 0.0078) and specificity (98.0 ± 1.8 versus 50.1 ± 0.8, **P = 0.0078). **c**, Experimental implementation of the CL algorithm included (1) continuous data recorded from *N* electrodes (1.1) and segmented with a sliding window (1.2); (2) a feature reduction preprocessing where the power spectral density estimate is calculated for each analysis window and for each electrode (2.1), and where a set of filters decompose the frequency content of the signal and reduce it to *M* coefficients (2.2) that are decorrelated from each other (2.3); (3) a feedforward ANN with two output classes, namely ‘pregrooming’ and ‘other behavior’, which processes the data for each electrode; and (4) automatic optogenetic stimulation following a threshold policy (>50% of the ANNs had to agree on a positive prediction of a grooming event). The orange shading shows a specific data window from electrode 1 and how it is processed in the different steps. Overall, this approach allowed the representation of the energy distribution envelope to be reduced by 99.99%, enabling real-time signal processing. Datapoints, single *Sapap3*-KO/PVCre mice; bars, means ± s.d. All statistical tests were Wilcoxon matched-pairs signed-rank one-tailed tests. Source data

**Extended Data Fig. 3:**
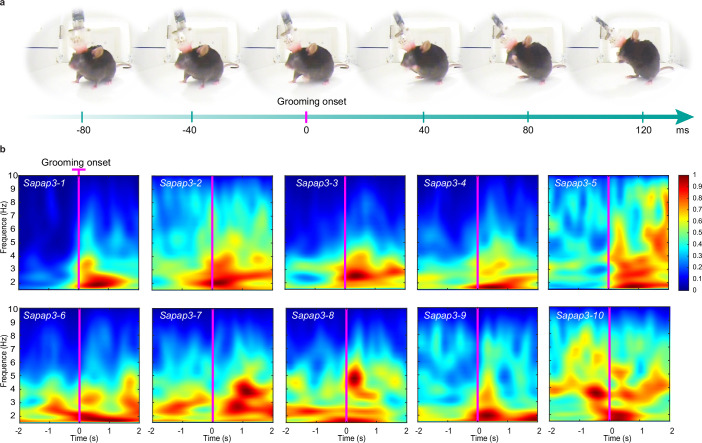
Low-frequency LFP signature in the OFC predicts grooming onset. (**a**) Example of consecutive image frames displaying grooming onset and the assignment of the exact time point labelled ‘grooming onset’ to the first frame showing the upwards locomotion of the anterior limbs towards the orofacial area to engage in a grooming bout. (**b**) Individual time-frequency wavelet spectrogram around grooming onset (-2 to +2 s peri-grooming onset) reveals an increase in low-frequency power in each recorded animal (n = 10 Sapap3-KO mice). Vertical magenta lines in each spectrogram indicate grooming onset as assigned in panel a. Each spectrogram represents the average normalised LFP activity of grooming events (*M* = 25 events, *SD* ± 5.25) for a single mouse extracted over different recording sessions; warmer colours represent higher energy.

To causally prove that striatal PVI recruitment is sufficient for grooming onset regulation, we sought a predictive biomarker to drive their activity before GO. To this end, we focused on one of the most prominent candidate regions implicated in compulsive-like behaviors, the OFC^3,31–33^. Its rodent homolog has also been shown to play a crucial neurophysiological role in the *Sapap3*-KO mouse model of compulsive-like behaviors^6,25,34,35^. We investigated grooming-related local field potential (LFP) activity in freely moving *Sapap3*-KO/PVCre mice (*n* = 10) by performing in vivo extracellular tetrode recordings in the lateral OFC (lOFC) (Supplementary Fig. 3). We manually annotated video frames of GO, which we defined as the first noticeable movement of front limbs engaging in a grooming event (Extended Data Fig. 3a). We selected grooming events with no preceding motor behaviors to exclude movement confounders. We contrasted the power spectral density of average pregrooming activity (−1 s to GO) with average resting activity (Fig. 3a). Despite the comparable stillness displayed by the animal during resting and pregrooming states, their corresponding power spectrum distributions differed, particularly within the delta frequency band (1–4 Hz) (Wilcoxon matched-pairs signed-rank test, *P* = 0.01). This low-frequency band power in pregrooming activity consistently increased towards GO (Fig. 3b). This ramping effect was characterized by an average rising point around 1 s before GO and average maximal power of around 2 Hz (Fig. 3c–e). The narrow temporal power increase in the delta range frequency preceding GO was observed in individual *Sapap3*-KO/PVCre mice (Extended Data Fig. 3b). As the average rising point occurred about 1 s before GO and the pregrooming maximal power fluctuates within the delta frequency band (1–4 Hz), this power increment embedded the predictive dimension required for driving PVI activity before GO by using CL neuromodulatory intervention. To that end, we developed a supervised learning method for automatically processing neural signals acquired across several tetrodes (Fig. 4) and predicted two possible output classes: either grooming (positive) or other (negative) behavior. To measure the probability of either event, we evaluated the two types of errors in a binary test (false positive and false negative errors) and the two correct results (true positive and true negative) (Fig. 4a). We then used these results to assess the performance of the algorithm by calculating sensitivity, specificity, precision and accuracy (Fig. 4b and Extended Data Tables 1 and 2). These four metrics were evaluated in a continuous experimental setup without the intervention of optogenetic stimulation. Using such an approach, our CL system achieved above-chance performance in predicting grooming activity from a cohort of *Sapap3*-KO/PVCre mice, comprising seven individuals (*n* = 7, 10 min each, with the algorithm making a decision every 200 ms) with an average percentage of 88.8 ± 11.8 sensitivity, 74.9 ± 11.2 precision, 98 ± 1.8 specificity and 94.2 ± 2.8 accuracy (mean ± s.d.). The methodological challenge of this CL design (Fig. 4c) was to develop a computationally lightweight LFP signal processing method for immediate feedback stimulation. Our technical innovation was thus to decompose unprocessed signals into a small representative matrix of coefficients reflecting the energy distribution of a given period. This was achieved by implementing triangular filter sets centered on the frequency band of interest, which allowed for capturing small fluctuations around a frequency band of interest while integrating broader information from adjacent frequency bands. This filter reduction step was essential to train and run a light feedforward artificial neural network (ANN) with one output layer giving two possible outcomes, one for pregrooming events and one for any other behavior, such as scratching, rearing, walking, standing and resting. Given the previously identified GO LFP signature, we designed a filter distribution for each mouse in the low-frequency range between 1 and 13 Hz (*f*-max mean = 10 ± 3.27) with seven filters. Taking advantage of such LFP filter decomposition, we drastically reduced the number of input variables for training to 0.01% of the original number of inputs; that is, by continuously processing 1 s of 32 acquisition channels sampled at 20 kHz, we reduced the input data dimension to only 35 features. LED stimulation was triggered if LFP activity recorded on at least 50% of the electrodes was classified as pregrooming. With such an approach, our CL system achieved above-chance performance to predict grooming activity for each animal and with an average of 10,800 decisions per mouse (12 trials of 3 min each, with the algorithm taking a decision every 200 ms) (Fig. 4b).

### CL prevention of compulsive-like grooming behavior

**Fig. 5:**
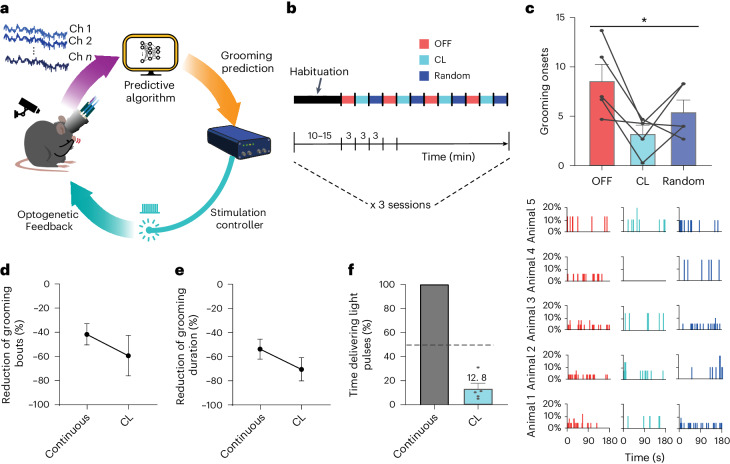
CL optogenetic intervention alleviates compulsive-like grooming in *Sapap3*-KO/PVCre mice. **a**, Schematic representation of the CL experimental paradigm for on-demand optogenetic intervention. OFC LFPs are acquired in *Sapap3*-KO/PVCre mice via chronic implants for neural activity recording and light stimulation. OFC LFPs are digitalized and processed using the novel online feature decomposition and reduction methods described above (purple arrow). If the supervised classification algorithm detected the low-frequency biomarker predictive of grooming onset through categorizing the processed OFC LFPs, a control signal (orange arrow) was sent to activate a stimulation controller to deliver blue-light pulses during 4 s. **b**, Illustration of the CL optogenetic stimulation protocol alternating ‘OFF’ (red), ‘CL ON’ (cyan) and ‘Random’ (blue) stimulation trials. **c**, The CL optogenetic stimulation reduced the average number of grooming onsets of *Sapap3*-KO/PVCre relative to OFF and randomized trials (top panel) (Page’s *L-*test for ordered alternatives, *H* = CL < randomized < OFF, *P = 0.02; GLMM, *P = 0.02). The histograms display the distribution of grooming onsets for each animal throughout the experimental period under different conditions (bottom panel). **d**,**e**, Percentage of reduction in grooming bouts (**d**) and in grooming duration (**e**) for continuous and CL conditions. **f**, Comparison of relative stimulation time between continuous and CL stimulation protocols. Data represent means ± s.e.m. Each graph depicts results of *n* = 5 *Sapap3*-KO/PVCre mice. Source data

**Extended Data Fig. 4:**
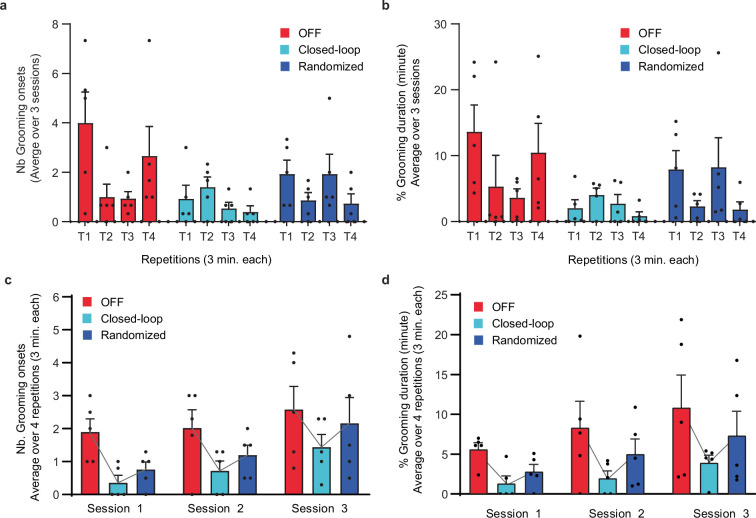
Intra- and inter-session comparison of OFF, closed-loop, and randomized optogenetic stimulation. (**a**) Detailed overview of the number of grooming onsets and the (**b**) percentage of grooming duration, segregated into individual repeated trials (T1-T4) of OFF (red), Closed-loop ON (cyan) and randomized (blue) optogenetic stimulation. Each dot represents the average of each individual mouse across three experimental sessions. (**c**) Detailed overview of the number of grooming onsets and the (**d**) percentage of grooming duration, segregated into sessions (1-3) and OFF (red), Closed-loop ON (cyan), and randomized (blue) optogenetic stimulation condition. Individual dots represent the average of each individual mouse across four different trials per session. All graphs depict the behaviour of n = 5 Sapap3-KO/PVCre mice. Data are presented as mean values + SEM. The grey line in panels (**c**) and (**d**) highlights the trend of average grooming number or onsets across the three stimulation protocols. Source data

**Extended Data Fig. 5:**
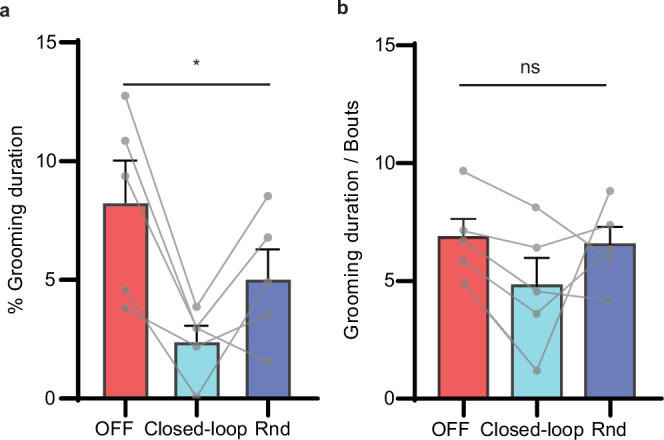
Closed-loop optogenetic stimulation effect on grooming duration. (**a**) Closed-loop optogenetic activation of PVI in the striatal regions receiving major lOFC input reduced the average percentage of grooming duration in Sapap3-KO/PV-Cre compared to ‘OFF’ and ‘randomized’ trials (Page’s L test for ordered alternatives, H=Closed-loop<randomized <OFF, L = 68, *P = 0.01). Grey dots and interconnecting lines represent the average of individual animals across trials and sessions. (**b**) The stimulation protocol did not affect the average duration of individual grooming events (s) in either the ‘OFF’, ‘Closed-loop’, or the ‘randomized ‘ condition (repeated measures one-way ANOVA, *P* = 0.9). Data are depicted as means ( + SEM) in the n = 5 Sapap3-KO/PV-Cre mice tested in the protocol. Source data

**Extended Data Fig. 6:**
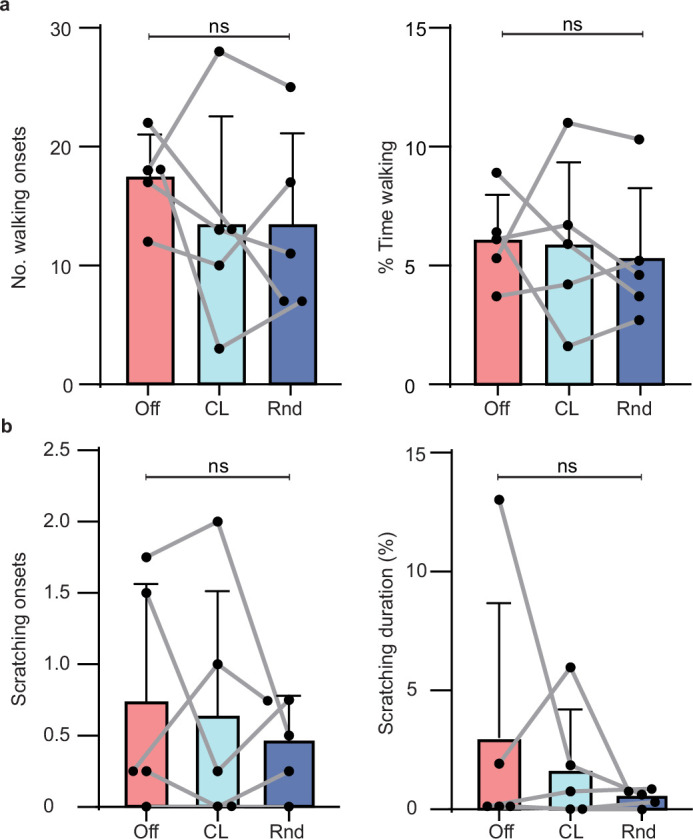
No evidence of change in scratching or walking with different stimulation treatments. Walking events during periods without stimulation, as well as during closed-loop or random stimulation, did not show evidence of difference in terms of (**a**) their number (M_Off_= 17.40 ± SD 3.578, M_CL_ = 13.4 ± SD 9.127, M_RND_ = 13.40 ± SD 7.66, Page’s L test for ordered alternatives, Closed-loop<Yoked<OFF, L = 57, P = 0.86) (left panel) and relative duration (M_Off_= 6.08 ± 1.89, M_CL_ = 5.88 ± 3.46, M_RND_ = 5.3 ± 2.95, Page’s L test for ordered alternatives, Closed-loop<Yoked<OFF, L = 61, P = 0.44) (right panel). (**b**) Similarly, scratching behaviour did not show evidence for a difference across these stimulation conditions regarding the number (M_Off_= 0.75 ± 0.81, M_CL_ = 0.65 ± 0.86, M_RND_ = 0.45 ± 0.33, Page’s L test for ordered alternatives, Closed-loop<Yoked<OFF, L = 59.5, P = 0.56) (left panel) and relative duration M_Off_ = 3.05 ± 5.63, M_CL_ = 1.72 ± 2.5, M_RND_ = 0.51 ± 0.35, Page’s L test for ordered alternatives, Closed-loop<Yoked<OFF, L = 59.5, P = 0.56) (right panel). Each data point represents one of the n = 5 Sapap3-KO/PV-Cre mice analysed across (a) four and (b) twelve independent 3-minute trials. The bars indicate the mean values ± standard deviations. Source data

Taking advantage of the real-time detection of LFP signal in the lOFC predicting grooming onset, we designed a CL approach in *Sapap3*-KO/PVCre mice expressing hChR2 (H134R)-mCherry. In this approach, the real-time prediction of GO would trigger an optogenetic controller to deliver on-demand light stimulation bilaterally to specifically recruit striatal PVI when GO is predicted (Fig. 5a). We designed a three-dimensional (3D)-printed homemade headstage for this experimental paradigm that allowed us to implant tetrodes in the lOFC and optic fibers bilaterally in the striatal areas receiving main input from the lOFC (Supplementary Fig. 2c–e). Similar to the continuous stimulation procedure, the experimental paradigm included a habituation stage of 10–15 min, followed by alternating 3-min trials of ‘OFF’, ‘CL’ and ‘randomized’ stimulation (12 trials in total) (Fig. 5b). During ‘OFF’ trials, no stimulation was delivered. During ‘CL’ trials, grooming events were predicted automatically online, which triggered the delivery of blue-light pulses for 4 s (5-ms pulses at 20 Hz and 10 mW). During ‘randomized’ trials, the number of light stimulations equalled the preceding ‘CL’ trial; however, they were assigned randomly across the 3 min. This protocol was repeated on three different days. We found that CL optogenetic stimulation significantly reduced GO in *Sapap3*-KO/PVCre mice (*n* = 5) compared with ‘OFF’ and ‘randomized’ trials (Page’s *L-*test for ordered alternatives, *H* = CL < randomized < OFF, *L* = 67, *P* = 0.02) (Fig. 5c). As in the continuous stimulation protocol, this effect was consistent across trials and sessions (GLMM–Type II Wald chi-square test: Chisq = 7.49, d.f. = 2, *P* = 0.02) (Extended Data Fig. 4a–d) and observed across all animals (Fig. 5c, bottom). The number of bouts and the grooming duration were reduced by 59.37% and 70.54%, respectively (Page’s *L-*test for ordered alternatives, *H* = CL < randomized < OFF, *L* = 68, *P* = 0.01; GLMM–Type II Wald chi-square test: Chisq = 12,292, d.f. = 2, *P* = 0.002) (Fig. 5d,e and Extended Data Fig. 5a). Thus, just as with continuous stimulation, CL optogenetic approach decreases the aberrantly high frequency of self-grooming events in *Sapap3*-KO animals to WT levels (Mann–Whitney *U*-test: *P* = 0.476). In line with results obtained in continuous optogenetic stimulation experiments, individual grooming sequences remained unaltered (Extended Data Fig. 5b). Just as continuous stimulation, our ‘CL’ approach specifically affected self-grooming behavior, while other behaviors, including walking as general behavior and hindpaw scratching as another form of repetitive behavior distinct from self-grooming, did not show evidence for a difference across stimulation conditions (Extended Data Fig. 6a,b). Finally, our CL stimulation was as efficient as the ‘continuous’ stimulation protocol in terms of reducing the number of grooming bouts (Fig. 5d) and grooming duration (Fig. 5e), but with a tremendous reduction of stimulation time by 87.2% (Fig. 5f).

## Discussion

By combining optogenetic neuromodulation with a CL stimulation protocol, we demonstrated in our study that the recruitment of striatal PVI before grooming onset was sufficient to regulate compulsive-like self-grooming behaviors in *Sapap3*-KO mice. We first showed that continuous optogenetic stimulation of the PVI network in the striatal areas receiving input from the lateral OFC immediately reduced the number of compulsive-like grooming events in *Sapap3*-KO mice to the level of matched WT controls while not affecting other types of behaviors. Building upon comprehensive, methodologically dedicated studies^36,37^ our optogenetic stimulation protocol was designed to avoid thermal heating. Although we have not replicated such technical assessment in this study, we are confident that our neuromodulatory effect is not due to thermal heating given that control *Sapap3*-KO/PVCre mice expressing a control virus without opsin do not decrease their grooming levels. Next, when seeking a neurophysiological predictor of compulsive-like self-grooming events to define the critical period for the recruitment of the PVI network, we detected an increase in the spectral power of the delta band activity in the lateral OFC, which shortly and consistently preceded grooming onset. We report transient LFP events in the OFC that predict compulsive-like grooming episodes. Using this predictor, we designed a CL approach based on an innovative supervised machine-learning protocol to recruit the striatal PVI network before compulsive-like grooming onset. Via this CL system, we showed that brief optogenetic PVI excitation upon grooming event prediction was sufficient to prevent their occurrence and, as a consequence, to specifically reduce compulsive-like self-grooming behaviors in *Sapap3*-KO mice. Taken together, within the scope of the chosen mouse model, the restriction to the neurobiologically valid phenotype of compulsive-like self-grooming, and the general caution that needs to be applied to low sample sizes in the context of experimentally challenging designs, we believe these results will be valuable for understanding the neurobiological mechanisms underlying repetitive behaviors; furthermore, they also open up new alleyways for designing therapeutic interventions for treating pathological repetitive behaviors.

Our study proposes that the temporal recruitment of striatal PVI shortly before the onset of compulsive-like behaviors is crucial for regulating their initiation. Indeed, our results showed that striatal PVI is essential in preventing the onset of compulsive-like self-grooming behaviors in *Sapap3*^−/−^ mice, presumably by inhibiting hyperactive MSN projection neurons^6,20^. These observations align with previous studies that showed the implication of striatal PVI during the suppression of prepotent inappropriate actions, regulation of choice execution or generation of complex motor sequences^16,38,39^. We observed that CL or continuous stimulation decreased grooming behaviors of *Sapap3*-KO to comparable levels observed in WT mice. These findings suggest that optogenetic PVI recruitment is particularly efficient to prevent inappropriately frequent, that is, compulsive-like grooming events. However, we cannot exclude that our stimulation protocols might have also altered normal grooming behavior given that, in the *Sapap3*-KO mice, normal and exaggerated compulsive-like grooming are phenomenologically indistinguishable. The crucial role of striatal PVI in regulating compulsive-like grooming in the *Sapap3*-KO mice is also supported by recent studies describing abnormal synaptic properties of striatal PVI in this animal model^40^ as well as by observations in other mouse models suffering from pathological repetitive behaviors where abnormally low PVI density in striatal areas was observed^23^.

However, the mechanisms by which the striatal PVI network could be recruited still need to be characterized. Interestingly, two recent studies have shown a coincidence in the emergence of delta oscillations in the prefrontal cortex and the recruitment of pyramidal cell assemblies^41,42^. In the context of our study, it would be interesting to investigate whether such recruitment of pyramidal cell assemblies occurs through the OFC delta oscillations observed here, which preceded compulsive-like grooming. Consequently, such cortical drive could allow for the temporally adjusted recruitment of the striatal PVI network, which in turn regulates the expression of compulsive-like grooming behaviors. This hypothesis is supported by the observation of a decreased spectral power in the low-frequency band in the OFC of *Sapap3*-KO compared with WT mice, including in the delta range^34^. Thus, a lower power of the LFP delta band in the OFC of *Sapap3*-KO mice may underlie impaired recruitment of downstream striatal PVI networks and, thus, a decreased regulation of compulsive-like self-grooming behaviors. Our findings also echo a recent study in OCD patients implanted with recording-capable DBS devices; the authors showed that LFP signals recorded in the striatum were co-occurring with OCD symptoms and detected a signature of interest in the delta band, as we observed in our study^43^.

Our findings also call for testing potential therapeutic strategies where striatal PVI could be specifically targeted in pathologies with compulsive behaviors. For instance, electrical DBS protocols have been used in severe, treatment-refractory OCD patients with good, but sometimes limited, efficiency in reducing compulsive symptoms depending on the brain structures targeted along the basal ganglia circuits^44–46^. Most efficient DBS protocols, with comparable clinical results, have targeted the associative domain of the anteromedial subthalamic nucleus or the anterior limb of the internal capsule^8,46–49^. However, improved stimulation protocols might leverage the potential of the associative striatum as DBS target structure^8,50^. Indeed, exclusive targeting of the associative striatum is neurosurgically easier to achieve than in smaller subthalamic nucleus or anterior limb of the internal capsule areas, where side effects can be observed due to closely located limbic and sensorimotor domains. To improve conventional continuous electrical DBS, cell-type-specific stimulation protocols are required. A recent study in which the authors demonstrated that they could specifically recruit PVI in the globus pallidus by using brief bursts of electrical stimulation confirmed such a possibility^51^. Building upon these findings and our results, it will be interesting to test such optimized DBS procedures in the context of pathological repetitive behaviors to recruit striatal PVI specifically.

Another interesting aspect of our study was developing an innovative supervised machine-learning approach for the automated delivery of on-demand optogenetic stimulation. The technical innovation of our approach for continuous processing of electrophysiological brain signals was to develop a decomposition stage for the drastic reduction of online-handled variables. Thus, the following signal processing stages require less computing power and allow for simpler architectures that could be implemented in general-purpose computers, as we did in our experiments. We believe that this methodology would be of great interest for exploiting neurophysiological signals in a real-time manner to reach greater temporal specificity, such as in adaptive DBS^51^ and, more generally, in brain–machine interface processes^52,53^. However, it should be mentioned that our machine-learning approach based on an ANN may present more implementation challenges compared with more classical alternatives, such as, for example, a threshold-crossing method. Our choice was based on the necessity to detect subtle changes of LFP pattern activity, essential for intervening before self-grooming onset. The predictive capability of this approach is crucial, especially since the peak in LFP biomarker power occurs only after grooming onsets—a scenario where threshold-crossing methods might be less effective. Our CL approach was as efficient in decreasing compulsive-like grooming as our continuous approach, with a reduction of the stimulation time by 87%, a crucial parameter in DBS therapy for battery saving. Even more interesting than this technical advantage is investigating potential long-term therapeutic effects such as recently observed using patterned neuromodulation protocols^51^. Finally, we believed that the implemented predictive approach might serve as a platform to further explore other brain targets for therapeutic purposes in a CL manner.

## Methods

### Mice

All experimental procedures were approved by the French Ministry of Higher Education, Research and Innovation (APAFIS reference nos. 1418-2015120217347265 and 31141-2021042017105235). Mice were maintained in a 12-h light/dark cycle with ad libitum food and water, maintaining recommended temperature at 20 °C to 26 °C (68 °F to 79 °F) and humidity at 40% to 60%. The *Sapap3*-KO mouse line was provided by G. Feng and A. M. Graybiel (Massachusetts Institute of Technology) (B6.129-*Dlgap3*^*tm1Gfng*^/J; Jackson Laboratory stock, catalog no. 008733) and backcrossed on C57BL/6 background strain (Jackson Laboratory) every five to ten generations. Heterozygous male and female *Sapap3*^+*/−*^ mice from this breeding were used at the age of 3–4 months to be crossed with mice from the (Pvalb)-Cre line. *Sapap3*^+/−^ were crossed with parvalbumin (PV) (*Pvalb*)-Cre line (B6;129P2-*Pvalb*^*tm1(cre)Arbr*^/J; Jackson Laboratory stock, catalog no. 008069; males and females, 3–4 months old; provided by A. Bacci, Paris Brain Institute, France) to obtain Cre-positive *Sapap3*^+/+^ and *Sapap3*-KO mice. Biopsies for genotyping were taken during weaning and at the end of all experimental procedures to verify the absence or presence of the Sapap3 and Cre-recombinase proteins, respectively, according to genotyping recommendations of the Jackson Laboratory (standard PCR assay protocol nos. 24678 and 27783 for PV-Cre and *Sapap3*-KO line, respectively). For experimental procedures, we used 17 male Sapap3^−/−^::PV-^Cre/wt^ (average initial age = 8.6 ± 1.4 months, average weight = 30.5 ± 3.7 g) and 5 age-matched male *Sapap3*^+/+^::PV-^Cre/wt^ (average initial age = 9.1 ± 1 months, average weight = 39.3 ± 4 g). Although data collection was not performed blind to the conditions of the experiments, the selection of animals was based solely on matched age and weight ranges and the expression of the overgrooming phenotype in *Sapap3*-KO mice. The group allocation was then decided according to animal genotype and/or the treatment previously administered to the animal, that is, the type of virus injected. Although no statistical methods were used to predetermine sample sizes, our sample sizes were selected for each experiment based on variance observed in previous experiments of a similar nature and practical considerations^25,54,55^.

### Viral vectors

Viral vectors expressing either an excitatory opsin and a fluorophore reporter (AAV5-hEF1a-dlox-hChR2(134 R)-mCherry(rev)-dlox-WPREhGHpA) or a fluorophore reporter only (AAV5-hSyn1-dlox-mCherry(rev)-dlox-WPRE-hGHpA) were purchased from the Viral Vector facilities of the Neuroscience Center Zurich (University of Zurich and ETH Zurich) and Addgene Viral Service facilities. Viral vectors were generated from Addgene plasmids catalog nos. 20297 and 50459, and titers were determined via fluorometric quantification (Neuroscience Center Zurich) or by real-time quantitative PCR combined with SYBR green technology (Addgene). Respective titers were >9.1 × 10^12^ vector genomes ml^−1^ (Neuroscience Center Zurich, opsin construct), >1.3 × 10^13^ vector genomes ml^−1^ (Neuroscience Center Zurich, control construct) and 1.4 × 10^13^ genome copies ml^−1^ (Addgene, opsin construct). Until stereotaxic injection surgeries, the viral vectors were aliquoted and stored at −80 °C until stereotaxic injection surgeries.

### Headframe implant

Homemade implants were designed and constructed around a transparent plastic core—a modified version of the Open Ephys flexdrive^56^ (Supplementary Fig. 2a). The implant was designed with SolidWorks 3D CAD software (Dassault Systèmes SolidWorks Corporation) and printed via stereolithography using the 3D printer Form2 (Formlabs) with clear resin. The plastic drive body core held eight independently mobile tetrodes and two fixed flat optic fiber stubs (Plexon Inc.) of 200/230 µm (core/core + cladding) inserted in a zirconia ferrule (Supplementary Fig. 2c–e). Tetrodes were made from 17.78-µm (0.0007 inches) Formvar-coated nichrome wire (A-M Systems), twisted (Tetrode Assembly Station, Neuralynx) and heated to form tetrodes. Tetrodes were fixed to laser-cut plastic springs (polyethylene terephthalate, 25 mm, Weber Metaux) and driven individually via miniature screws (0.7 M Micro-modele). Electrodes were attached to an electrode interface board (Open Ephys Production Site) with gold pins (Neuralynx). Individual electrodes were gold-plated to an impedance of 200–350 kΩ. Ground connections were made using 50.8-µm (0.002 inches) Formvar-coated tungsten wires (A-M Systems). The fully assembled implant (drive body, cap and cone) weighed less than 3 g.

### Surgical procedures

Each mouse received an injection of analgesics (buprenorphine 0.1 mg kg^−1^; subcutaneously) 30 min before deep anesthesia (induction at 2.5% isoflurane; maintenance during surgery at 0.9–1.2% isoflurane). The animals were mounted gently into a digitally equipped stereotaxic frame (David Kopf Instruments) via ear bars adapted for mouse stereotaxic surgeries. The animal’s temperature was maintained at 37 °C through a heating pad coupled to an anal sonde. Scalp hair was removed using depilation cream (Veet) diluted with sterile 0.9% saline, and the skin was disinfected three times with 70% ethanol and betadine solution (Vétédine, Vétoquinol) before skin incision. Skull and skin were maintained humid during the entire surgery using sterile 0.9% saline. Four small burr holes were drilled around the perimeter of the exposed skull surface to accept steel anchor screws. Two additional small burr holes were drilled behind the estimated implant volume to insert two separate ground wires. For all mice, craniotomies and durotomies (1.0 mm diameter) were made bilaterally at the following coordinates: AP = +1.0 mm, ML = +1.5 mm. For mice with in vivo tetrode recordings in the lOFC, an additional cranio-/durotomy (0.8 mm diameter) was performed at the following coordinates: AP = +2.8 mm, ML = +1.5 mm. The brain surface was maintained humid with sterile 0.9% saline throughout viral injections until headframe implantation. Adeno-associated virus constructs were injected bilaterally at a constant rate of 50 nl min^−1^ (0.4 µl per site) into the striatum (DV = −2.4 mm, AP = +1.0 mm, ML = +1.5 mm) using a motorized micro pump (Legato 130, KD Scientific) with a precision syringe (Hamilton Gastight Series, catalog no. 1701, 10 µl) and respective needles (Hamilton, 33Ga, bevelled end). Before virus injection, the needle was lowered to DV = −2.5 mm and retracted to DV = −2.4 mm immediately, where the needle was allowed to settle for 2 min. After injections, the needle was maintained in the same position for 10 min, then retracted by 300 µm (DV = −2.1) and maintained at that position for another minute. Afterwards, the needle was removed slowly from the brain. After each injection, to exclude needle clogging during intracranial procedures, correct flux was tested by visually checking the continuous formation of a droplet using the same injection speed as during intracranial injections. A drop of surgical lubricant was applied to each cranial opening. The implant was lowered carefully so that the bottom polyamide tubes containing either tetrodes or smaller tubing to hold the optic fibers touched the brain surface. The implant was fixed to the skull and screws with dental acrylic (Jet Denture Repair, Fibred Pink powder + liquid, Lang Dental). Ground wires were formed into tiny loops inserted below the skull to make surface contact with the brain. Ground wires were then fixed to the implant frame and connected to the electrode interface board. Optic fibers were lowered carefully to a depth of 2 mm into the brain and glued into position in the opening provided in the electrode interface board. Tetrodes were advanced individually directly after surgery in the OFC (DV = 1.0 mm) or the striatum (DV = 1.5–2 mm) during the following 3–5 postoperative days. A protective 3D-printed cap was screwed to the implant to keep out bedding debris. Animals were injected with analgesics (buprenorphine, 0.1 mg kg^−1^; subcutaneously) directly after surgery and every 12-h postsurgery on the following day; they were monitored closely until awake in a heating chamber set to 37 °C. Animals were then placed into a clean home cage equipped with materials adapted for implanted animals (for example, no hanging food grids to avoid bumping the implant). Animals were closely monitored postsurgery twice a day, including veterinary care, taking into account changes in weight, body score, nesting and overall locomotor activity as indicators of surgery recovery.

### Experimental setup

The experimental setup consisted of a homemade arena (290 mm × 270 mm) in which the mouse was allowed to move freely. The setup structure included passive commutators for electrophysiology and optic stimulation, synchronization LEDs, and two cameras (704 × 576 resolution; 25 frames per second (fps)) installed at opposite walls to allow for complementary views. After surgical recovery, the animals were habituated for 3 days to human handling, the electrophysiology acquisition system and the fiber patch cable tethering. The animals were left in the arena with ad libitum food and water for two nights (6 p.m. to 8 a.m.) of additional habituation to the setup. At the beginning of each experimental session, the mice were carefully tethered and habituated to the arena for 10–15 min. Any noises and vibrations were avoided during experimentation. No water or food was provided during the experimental sessions. After each experiment, the behavioral apparatus was cleaned using a disinfectant cleaning spray containing 55% ethanol, washed with soap and water, and dried.

### Behavioral assessment

Manual scoring was performed using a freely available video scoring software (Kinovea, v.0.8.15; https://www.kinovea.org/). Self-grooming activity was assessed by quantifying duration, number of grooming events and the percentage of grooming time. Grooming onset was defined as when the mouse started lifting its front paws to groom. The end of grooming was defined as the time point when the mouse stopped grooming for at least 1 s or when the grooming behavior was interrupted by another behavior. We discarded grooming-like phases of less than 1-s duration. Regarding the percentage of reduction in grooming bouts (Fig. 5d) and duration (Fig. 5e) for the CL condition, we considered the condition without stimulation (OFF) as our reference baseline of grooming activity. We then calculated the proportion of reduction in grooming activity in the CL condition as follows:

$${\mathrm{Reduction}}\; {\mathrm{of}}\; {\mathrm{grooming}}\; {\mathrm{bouts}}\left( \% \right)=100-\frac{{\mathrm{Gbout}}_{\mathrm{CL}}\times100}{{\mathrm{Gbout}}_{\mathrm{OFF}}}$$

$${\mathrm{Reduction}}\; {\mathrm{of}}\; {\mathrm{grooming}}\; {\mathrm{duration}}\left( \% \right)=100-\frac{{\mathrm{Gduration}}_{\mathrm{CL}}\times 100}{{\mathrm{Gduration}}_{\mathrm{OFF}}}$$

with $\mathrm{{{Gbout}}_{{OFF}}}$ and $\mathrm{{{Gduration}}_{{OFF}}}$ being the average grooming bouts and average grooming duration of the three recording sessions in our condition OFF, and $\mathrm{{{Gbout}}_{{CL}}}$ and $\mathrm{{{Gduration}}_{{CL}}}$ the corresponding average values in our CL condition.

We implemented an inter-rater control to validate the manual annotation of video frames. Three naïve raters that were trained previously by an expert rater to recognize grooming using an instructional video were assigned to annotate five recording sessions. Each 36-min video corresponded to a *Sapap3*-KO mouse and a single session from the CL experiments (60 blocks of 3 min each with 90 grooming events in total). We then calculated the error percentage per trial in the CL session between the raters as follows:

$${\mathrm{Error}}\,(\%) \,=\frac{{\mathrm{grooming}}_{\mathrm{expert}}-{\mathrm{grooming}}_{\mathrm{naive}}}{{\mathrm{grooming}}_{\mathrm{expert}}} \times100$$

The three raters consistently achieved an acceptable minimal error for the percentage of time spent grooming (${{{M}}}_{{\rm{s}}1}=8.75\pm 4.37,{{{M}}}_{{\rm{s}}2}=7.018\pm 3.107,{{{M}}}_{{\rm{s}}3}=7.79\pm 4.618$) and for the grooming events (${{{M}}}_{{\rm{s}}1}=6.88\pm 4.6,{{{M}}}_{{\rm{s}}2}=5.804\pm 7.599,{{{M}}}_{{\rm{s}}3}=8.122\pm 7.055$), confirming the level of agreement among raters.

We annotated other behaviors, such as walking, rearing, sniffing, resting, stretching, heading up or freezing. Walking was defined as forward locomotion of the animal on all four paws. Hindpaw scratching was defined as rhythmic movement of the hind limbs interacting with more rostral parts of the body. The targeted body parts varied between individuals in snout, area around the eyes, upper forehead, neck, between shoulders and on the back^57,58^. The neural activity during these behaviors was used to form a database for our supervised learning algorithm. For the analysis of the LFPs, we assessed resting behaviors. These were defined as behavioral activity in which the animal remained awake but did not engage in active behaviors such as walking, climbing, scratching, rearing and grooming. The head position was in extension of the spine, with the four limbs touching the ground and the mouse in a relaxed posture, that is, not hunched over with exaggerated breathing as in freezing behavior; the ears were not pulled back, and the eyes were open normally.

To quantify global motion in mice during ON and OFF stimulation, we used DeepLabCut (v. 2.2.1, with CUDA Toolkit v.11.0 and Tensorflow v.2.3.0)—an open-source Python toolkit for tracking body parts in videos^59^ following the protocol published in ref. ^60^. In summary, the DeepLabCut toolbox was used to extract frames from video recordings, manually label ten body parts of interest (snout, left ear, right ear, front center, middle back/hump, tail base, right paw, left paw, right limb and left limb) and train a deep-learning model to track the body parts automatically. The recording design included two facing cameras at the level of the animal, simultaneously recording the inside of the experimental setup from opposite directions. We annotated 60 frames per video and per camera (18 videos in total) from 6–13-min videos. For the training process, 95% of the images were used to create a training database. A ResNet-50-based neural network^61,62^ was used with 500,000 iterations. Validation was performed with a *P* cutoff value of 0.7, resulting in an average test error of 6.8 and 4.53 pixels and an average training error of 2.6 and 2.5 pixels for camera 1 and camera 2, respectively (image size was 704 × 576 pixels). Subsequently, this trained network was applied to analyze 1-h videos for the assessment of activity in the OFF/ON and CL protocols.

To estimate the global activity of the animal from the videos, the *x* and *y* coordinates of the tracked body parts obtained with DeepLabCut were processed with Matlab custom scripts (Matlab v.2022b). Within each video frame, we used the tracked body parts to draw a polygon representing the contour of the body. We calculated its centroid, which we defined as the body center. During the experimental period, the camera that was the farthest from the subject was chosen as it captured more body parts and thus a better contour of the animal’s body. The instantaneous speed of the body center was determined between frames (25 fps) by deriving the body center mark over time. We applied a median filter (order *N* = 21) to the instantaneous speed data and calculated the s.d. of the recording, which we use as a threshold value to distinguish between low and high locomotion, as we observed that this threshold separates well instances where mice are relatively stationary or do not move much. If more than three body parts were not detected by any camera, the frames were discarded and marked as NA (not applicable). These represent instants when tracing the polygon is unreliable. They correspond to situations where it was challenging to detect the animal because of its position in the camera’s field of view. We calculated the percentage of experimental time in which the animal exhibited low and high activity according to video analyses, as well as the percentage of the time that was marked as NA.

### Optogenetics

Bilateral optogenetic neuromodulation in the striatal area receiving lateral orbitofrontal cortical projections was conducted at least 2 weeks after stereotaxic viral injections, to allow for sufficient recovery and viral expression. The implanted striatal fiber stubs were connected to optical patch cables (200-µm core diameter and 20 mm length with 0.66 numerical aperture) via mating ferrules in a zirconia sleeve. Optical patch cables were connected to blue-light LED source modules (465 nm) mounted on magnetic LED commutators. The light stimulation patterns were preprogrammed using Radiant Software (Plexon Inc.). Before experimentation, the output power was measured from the optical fiber tips with a light power meter (Thorlabs PM100D with S120C sensor) and calibrated to 10 mW. Unless otherwise stated, all devices for optogenetic modulations were purchased from Plexon Inc.

#### Continuous optogenetic stimulation

Each experimental session lasted 30 min and included ten interleaved OFF (no light stimulation) and ten ON trials (10 mW, 5-ms train light pulses at 20 Hz) of 3 min. This represents 10% stimulation duty cycle, which is within the range that minimizes the potential for tissue thermal heating and damage according to refs. ^36,37^. Each experimental session lasted 30 min and included five interleaved OFF (no light stimulation) and ON trials (10 mW, 5-ms train light pulses at 20 Hz) of 3 min each.

#### CL optogenetic stimulation

Each experimental session lasted 36 min. In four repetitive cycles, we alternated OFF trials, trials with CL stimulation and with randomized stimulation, each lasting 3 min. A custom Matlab (R2017b) algorithm triggered the light stimulation on demand for the CL trials. Each activation lasted 4 s (10 mW, 5-ms pulses at 20 Hz). In a randomized trial, the same number of stimulations delivered in the previous CL trial was pre-assigned randomly (rand function) by the algorithm.

### Data acquisition and analyses

#### Electrophysiology

We performed extracellular recordings in awake, freely moving mice using tetrodes attached to a 32-channel connector in a mechanically adjustable drive. All signals were amplified, multiplexed and digitalized at a sampling frequency of 20 kHz using the Intan hardware acquisition system (RHD2000 USB Interface Board with the RHD 32-Channel Headstage, Intan Technologies) and either Intan Software (RHD USB Interface Board software) or Open Ephys GUI^63^. Recordings were analyzed offline. We used a custom Matlab code to record with Intan Libraries for the CL experiments. A blinking LED and a digital input activated simultaneously were used to synchronize video and extracellular recordings.

#### LFP analysis

Neural signals were synchronized with video recordings via transistor-transistor logic signals for LFP recordings. The spectral content of the LFP signals was analyzed using custom Matlab routines, the Chronux^64^ Matlab package (http://chronux.org) and the Matlab Signal Processing toolbox. For Fig. 3 and Extended Data Fig. 3, we decreased the broadband data sample rate by 40 and then low-pass filtered the data using a tenth-order Butterworth filter with a cutoff frequency of 10 Hz. We performed continuous wavelet transformation around grooming events (4 s before and after onset). We used the Morse wavelet with asymmetry parameter (*γ*) = 3 and a time-bandwidth product = 60.

#### Spike analysis

Spike sorting was performed offline using the valley-seeking prevalent method in Offline Sorter (v.3.3.5, Plexon Inc.), followed by visual screening. Each set of spike clusters was compared for cross-correlogram features and spike waveform. The spikes of each putative unit were assessed qualitatively regarding ISI-waveform, spike amplitude and waveform consistency. To quantify the effect of light, we used NeuroExplorer (v.4, Nex Technologies) rate histogram to display the firing rate versus time with a bin size of 0.05 s.

### Feature selection and supervised classification

#### Dataset creation

We created a dataset of independently extracted samples (1-s windows) of OFC activity of two categories, either pregrooming onset events (between −2 s to grooming onset) or other types of behavior (walking rearing, resting and so on). The dataset was extracted from LFP recordings in *Sapap3*-KO freely moving mice from five 1-h recording sessions for each experimental animal on different days. Each dataset contained a minimum of 100 events. The dataset was shuffled and split randomly into a training dataset (70%) and a test dataset (30%).

#### Feature reduction and supervised machine learning

To obtain the predictive value of low-frequency LFP components in the OFC related to the animal’s behavioral state (‘pregrooming’ and ‘other behavior’), we designed a preprocessing procedure for feature reduction that captures small changes in the energy distribution in a particular frequency band over time. A set of triangular filters predefined by a symmetrical distribution around the principal frequency of interest *F* (Fig. 3e) within the range from *f*_A_ to *f*_B_ is chosen to represent the shape and distribution of energy of the LFP biomarker in the frequency domain (in our case, *f*_A_ = 1, *f*_B_ = 10 ± 3.27, *N* = 5). The filter distribution is configured to have gradually decaying information concerning the portion of the spectrum to which they are applied. Indeed, the set of filters is predefined around a principal frequency of interest, allowing the detection of small changes around any frequency of interest, *F*, while integrating broader information from adjacent frequency bands. Since the features extracted depend on the distribution of the filter set, the distribution function and its sampling are suited to detect a variety of neural signatures for which a change in energy at a specific frequency band is observed. For each analysis window (subsegments of 1 s), we calculate the power spectral density estimate, and the set of filters is applied to obtain a vector of *M* coefficients whose values are furthermore decorrelated using the discrete cosine transformation. After *P* iterations, we create a matrix of *M* *×* *P* decorrelated coefficients (*M* = 7, *P* = 5) that reflect the desired energy distribution in a period.

The feature matrix constituted the input for an ANN with a minimal architecture: one input layer with 35 neurons, two hidden layers, and an output layer with two neurons for each category, one for pregrooming behavior and the other for a collection of different types of behavior. Since each electrode was processed individually, we set a threshold policy to decide whether the output values of the neural network corresponded to a pregrooming event or not; only if more than 50% of these detections pointed to a pregrooming event it was counted as such.

#### CL implementation and assessment

For a CL implementation of the algorithm, extracellular signals in the OFC from freely moving mice were continuously acquired through 32 chronic drive implant recording channels (20 kHz sampling frequency), amplified and digitalized. The data were segmented with a sliding window with 200-ms time-shift and the power spectral density estimate is calculated for each analysis window and for each electrode to be treated by the feature reduction and machine-learning stage.

#### Optogenetic stimulation feedback

When put into practice in the CL experiments, one positive outcome of pregrooming classification would trigger the optogenetic stimulator for 4 s—a period in which the algorithm would not make any further decisions. For each outcome of the ‘other behavior’ class, the stimulator would remain off, and the tests would continue every 200 ms.

To estimate the algorithm’s performance in a CL-like scenario, we tested the algorithm offline on continuous 10-min recordings per mouse, from *n* = 7 *Sapap3*-KO/PVCre mice, with decisions made by the algorithm at 200-ms interval bins. In this test dataset, there was no optogenetic stimulation provided, allowing for an assessment of the algorithm’s predictive accuracy for grooming behaviors, independent of neuromodulatory influences. We defined the ‘early detection period’ (Ep) as the time window between −2 s to grooming onset to half-duration of the grooming event. True positive (TP) classifications were the predictions or light stimulations that fell within the Ep. False positives (FP) were all the other stimulations that did not fall inside the Ep. True negative (TN) results are the other behavior predictions outside Ep and grooming events and false negatives (FN) are all missed grooming events where no grooming prediction falls into the Ep as summarized in Supplementary Table 1.

In the test dataset, on average, recordings comprised 12.9 ± 5.1 grooming bouts, with a total of 50.3 s ± 24.4 of grooming Ep activity. To contrast our results with that of a pseudorandom classifier, we used the same behavioral data collected in the previous experiments test dataset (*n* = 7 *Sapap3*-KO/PVCre mice) and implemented a uniform pseudorandom number generator (1 for grooming and 0 for other behavior) with the same result rate (200 ms). For each mouse’s dataset, we ran the pseudorandom algorithm five times and reported average metric results. We calculated the accuracy (the proportion of all correctly categorized instances out of the total number of all correct and incorrect classifications), precision (proportion of true grooming predictions out of all grooming predictions; false and true), sensitivity (correct classification of LFP activity associated with the onset of self-grooming) and specificity (correct detection of the LFP signal associated with behaviors other than grooming) for both algorithms and each 10-min trial as described in Supplementary Table 2.

### Histology

After experimental procedures, electrolytic mark lesions were made to confirm the localization of tetrode recording sites. Animals were anesthetized as described above, and 5 µA constant current was delivered to each electrode for 20 s using an isolated current stimulator (DS3, Digitimer Ltd). After 72 h, mice were anesthetized via an intraperitoneal injection of pentobarbital (200 mg kg^−1^) and perfused transcardially with 30 ml of 4 °C 0.9% sodium chloride solution followed by 60 ml of 4 °C 4% paraformaldehyde in 0.1 M phosphate buffer. Brains were postfixed in the same paraformaldehyde solution overnight at 4 °C, briefly rinsed three times in 1× PBS, and progressively dehydrated for cryosectioning by incubations in 15% and subsequently in 30% sucrose solution in 1× PBS for 24 and 48 h, respectively. Next, brains were embedded in the OCT compound and sectioned into six series of 40-µm coronal sections (Microm HM, catalog no. 560, Thermo Scientific) into 1× PBS containing 0.1% sodium azide. Prefrontal sections were stained with a freshly filtered 1% cresyl violet solution and analyzed for the location of marked lesions under a brightfield microscope. Tetrode recording sites that were detected slightly outside of the lOFC were excluded from LFP analyses. Immunofluorescence stainings were performed on the striatal sections of one series in 4 °C solutions on an orbital shaker. Concretely, sections were washed three times for 10 min in 1× PBS, followed by three washes of each 10 min in 1× PBS containing 0.1% Tween 20 and 0.2% Triton-X. Sections were next blocked for 2 h in 5% normal goat serum in 1× PBS containing 0.1% Tween 20 and 0.2% Triton-X. Subsequently, sections were incubated in the same blocking buffer containing anti-red fluorescent protein (RFP) antibody (polyclonal rabbit anti-RFP, Rockland, catalog no. 600-401-379, catalog no. 35634; dilution, 1:1,000) on an orbital shaker at 4 °C overnight. Sections were then washed three times for 10 min in 1× PBS with 0.1% Tween 20 and 0.2% Triton-X and incubated for 2 h in a blocking buffer containing a secondary antibody (polyclonal anti-rabbit Cy3-conjugated AffiniPure, produced in goat, catalog no. 106489; dilution, 1:400). Afterwards, sections were washed twice for 10 min in 1× PBS with 0.1% Tween 20 and 0.2% Triton-X, followed by one wash for 10 min in 1× PBS. Next, sections were incubated for 2 min and 30 s in 4,6-diamidino-2-phenylindole solution (10 µg ml^−1^), washed three times for 10 min in 1× PBS and mounted in 0.1 M phosphate buffer onto Superfrost Plus slides and coverslipped using a fluorescence-protecting medium (Fluoromount, Sigma Aldrich). A selection of sections was incubated in the same buffer with an additional anti-parvalbumin antibody (polyclonal guinea pig anti-PV GP 72, Swant; dilution, 1:5,000) to verify viral infection of specifically parvalbumin-positive cells. The secondary antibody solution also contained polyclonal anti-guinea pig Alexa488, produced in goats (Invitrogen, catalog no. 145863; dilution, 1:400). All striatal sections were imaged using a slide scanner (Axio Scan.Z1, Zeiss), and the sections were analyzed and mapped using ZEN software (Zeiss) and a mouse brain atlas^65^.

### Statistical analyses

All statistical analyses using R were conducted using R v.4.1.1 (R Development Core Team, 2021) and those using Prism were conducted using v.8.0.1 (GraphPad Software Inc.). All levels of statistical significance were set at *P* < 0.05.

#### Continuous stimulation analyses

We applied nonparametric statistical testing using Prism to assess the effect of optogenetic neuromodulation in our continuous stimulation experiments. For within-animal comparisons, we applied Wilcoxon matched-pairs signed-rank tests (paired, nonparametric test, two-tailed, confidence level 95%) and for comparisons across animal groups, we applied Mann–Whitney testing (unpaired, nonparametric test, comparing ranks, two-tailed, confidence level 95%). In addition to evaluating the main effect of ‘treatment’, we assessed the consistency of this effect across trials and sessions using a GLMM. Before conducting GLMM analyses with Poisson distribution, we verified all GLM assumptions to ensure that the Poisson regression accurately fitted the data using the R package DHARMa (v.0.4.6)^66,67^. This package applies a simulation-based approach to create interpretable scaled residuals for GLMMs, including Poisson GLM^67^. Concretely, linearity, uniformity and Poisson distribution of residuals were checked using the QQ-plot of residuals and statistically tested using a Kolmogorov–Smirnov test (*D* = 0.048, *P* = 0.47). Absence of autocorrelation was tested using Durbin–Watson test (DW = 2.51, *P* = 0.43) and the dispersion test on the variance-to-mean ratio (dispersion = 0.95, *P* = 0.96) did not indicate under- or overdispersion of the count data. Taken together, assumption testing confirmed the applicability of a GLMM with Poisson distribution to both the continuous and the closed-stimulation data in our study. Hence, a GLMM using the Poisson family was fitted to explain the ‘number of grooming’ by the different ‘treatments’. Hereby, the model included ‘MouseID’, ‘Trial’ and ‘Session’ as random effects. ‘Trial’ random effect was nested to ‘Session’ random effect to appropriately account for the trial effect within each session. The significance of the main effects of continuous ON/OFF stimulation (‘treatment’) was assessed based on Type II Wald chi-square tests. Please note that the ‘glmmTMB’ function (from glmmTMB package v.1.1.4) was used to model the GLMM because it is intended to handle zero inflation, unlike the glmer function. Finally, we fitted a linear mixed model to explain also the grooming duration by continuous ON/OFF stimulation (‘treatment’). Again, the model included ‘MouseID’, ‘Trial’ and ‘Session’ as random effects. The ‘Trial’ random effect was nested to ‘Session’ as random effect. The square-root transformation was used on the grooming duration response variable to improve the model assumptions of linearity, normality and constant variance of residuals. The significance of the main effects of ON/OFF treatment was assessed based on Type II Wald chi-square tests from the ‘Anova’ R function (package ‘car’ v.3.1-0 with ‘Anova’ function in R).

#### Analyses of the classification algorithm

To assess the performance of our supervised classification algorithm compared with a pseudorandom classification algorithm, we applied paired *t*-tests for each performance parameter, that is, accuracy, sensitivity, precision and specificity, after previous verification of the normal distribution of the data.

#### CL stimulation analyses

To assess the statistical evidence for the increase in the ordinal ranks between three ‘treatments’ (CL, Yoked and OFF) in terms of the number of grooming, the repeated measure trend test—the nonparametric Page’s *L*-test for ordered alternatives^68^—was used with the PageTest R function in DescTools package (v.0.99.46), where the ordered alternative hypothesis H1, m_ClosedLoop<m_Yoked<m_Off, was tested against the null hypothesis H0, m_CL = m_Yoked = m_Off. Hereby, values were averages by animal and session. To assess consistency of main effect of ‘treatment’ across trials and sessions, we also fitted a GLMM using the Poisson distribution family to explain the ‘number of grooming’ and ‘grooming duration’ by the different stimulation ‘treatments’ (CL, Yoked and OFF). The model included MouseID, Trial and Day as random effects. The random trial effect was nested to day random effect to appropriately account for the trial effect within each session. A square-root transformation was used on grooming duration as a response variable to improve the model assumptions of linearity, normality and constant variance of residuals. Just as for continuous stimulation analyses, before conducting GLMM analyses with Poisson distribution, we verified all GLM assumptions to ensure that the Poisson regression accurately fitted the data using the R package ‘DHARMa’ (refs. ^66,67^). This package applies a simulation-based approach to create interpretable scaled residuals for generalized linear mixed models, including Poisson GLM^67^. Compliance with the model assumptions was confirmed with the QQ-plot of residuals and by the Kolmogorov–Smirnov (*D* = 0.065, *P* = 0.415), Durbin–Watson (DW = 1.819, *P* = 0.1278) and dispersion tests (dispersion = 1.25, *P* = 0.208). Taken together, assumption testing confirmed the applicability of a GLMM with Poisson distribution to the CL stimulation data. ‘Treatment’ effects were obtained through Type II Wald chi-square test. We then used the ‘emmeans’ package v.1.8.2 in R^69^ to conduct a two-sided Tukey’s honest significant difference test. *P* values resulting from the post hoc tests were obtained using Kenward–Roger’s approximation for degrees of freedom and were adjusted to control for the false discovery rate due to multiple comparisons.

Finally, to compare average grooming bout durations in the OFF, CL and Yoked stimulation conditions, we performed a one-way analysis of variance (Prism, GraphPad v.8.0.1).

### Reporting summary

Further information on research design is available in the Nature Portfolio Reporting Summary linked to this article.

## Online content

Any methods, additional references, Nature Portfolio reporting summaries, source data, extended data, supplementary information, acknowledgements, peer review information; details of author contributions and competing interests; and statements of data and code availability are available at 10.1038/s41593-024-01633-3.

### Supplementary information

Supplementary Information Supplementary Figs. 1–3 and Tables 1 and 2.

Reporting Summary

### Source data

Source Data Fig. 1 Statistical source data.

Source Data Fig. 2 Statistical source data.

Source Data Fig. 3 Statistical source data.

Source Data Fig. 4 Statistical source data.

Source Data Fig. 5 Statistical source data.

Source Data Extended Data Fig. 1 Statistical source data.

Source Data Extended Data Fig. 2 Statistical source data.

Source Data Extended Data Fig. 4 Statistical source data.

Source Data Extended Data Fig. 5 Statistical source data.

Source Data Extended Data Fig. 6 Statistical source data.

## Figures and Tables

**Extended Data Table 1:**
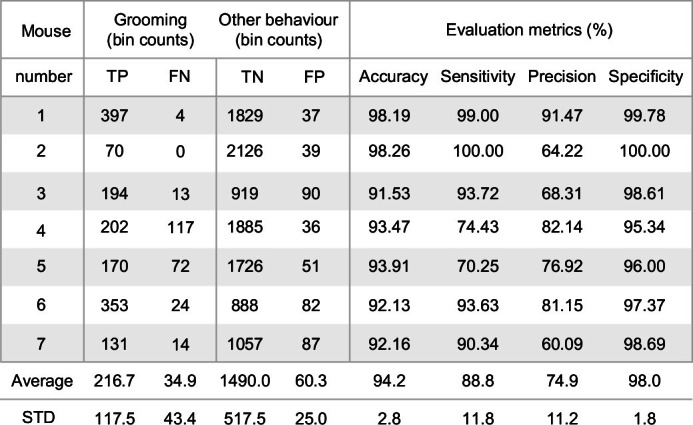
Closed-loop algorithm performance across a cohort of Sapap3-KO/PV Cre mice

Performance metrics for our closed-loop algorithm performed offline in Sapap3-KO/PVCre mouse (n = 7 mice in total). Each row represents the results of an individual mouse. Each experiment consisted of a 10-minute recording per mouse (without stimulation), with a decision made at 200-ms interval bins. On average, recordings contained 12.9 ± 5.1 grooming bouts. The results display the number of bins detected that were either True or False per category (‘Grooming’ or ‘Other behaviour’). Abbreviations: TP (True Positives), FN (False Negatives), TN (True Negatives), FP (False Positives), SD (Standard Deviation), nb (number).

**Extended Data Table 2:**
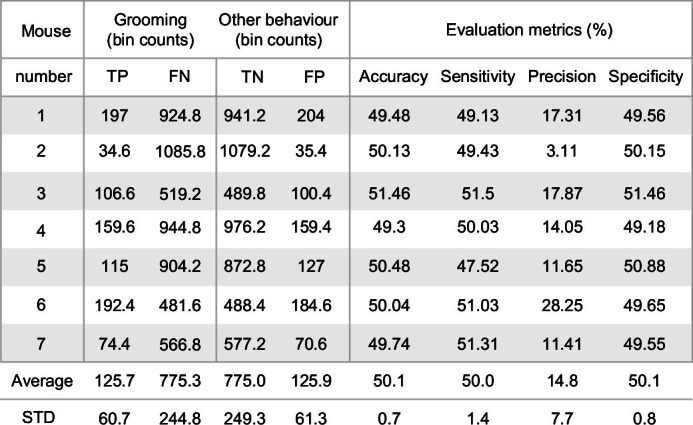
Random algorithm performance across Sapap3-KO/PV Cre mice

Comparative analysis of key performance metrics on the same recordings than in Extended Data Table 1, but from a random algorithm. Abbreviations: TP (True Positives), FN (False Negatives), TN (True Negatives), FP (False Positives), SD (Standard Deviation), nb (number).

## Data Availability

The data supporting this study’s findings are available in the publicly accessible repository OSFHOME via 10.17605/OSF.IO/KDMJT. Source data are provided with this paper.

## Code availability

All custom MATLAB code generated in this work is openly available in the repository GitHub via https://github.com/LizbethMG/2024_Mondragon-Gonzalez_NatureNeuroscience.

## References

[1] Gunaydin LA, Kreitzer AC (2016). Cortico-basal ganglia circuit function in psychiatric disease. Annu. Rev. Physiol..

[2] Corbit LH, Nie H, Janak PH (2012). Habitual alcohol seeking: time course and the contribution of subregions of the dorsal striatum. Biol. Psychiatry.

[3] Chamberlain SR (2008). Orbitofrontal dysfunction in patients with obsessive-compulsive disorder and their unaffected relatives. Science.

[4] Anticevic A (2014). Global resting-state functional magnetic resonance imaging analysis identifies frontal cortex, striatal, and cerebellar dysconnectivity in obsessive-compulsive disorder. Biol. Psychiatry.

[5] Joel D, Doljansky J, Schiller D (2005). ‘Compulsive’ lever pressing in rats is enhanced following lesions to the orbital cortex, but not to the basolateral nucleus of the amygdala or to the dorsal medial prefrontal cortex. Eur. J. Neurosci..

[6] Burguière E, Monteiro P, Feng G, Graybiel AM (2013). Optogenetic stimulation of lateral orbitofronto-striatal pathway suppresses compulsive behaviors. Science.

[7] Davis GL (2021). Ketamine increases activity of a fronto-striatal projection that regulates compulsive behavior in SAPAP3 knockout mice. Nat. Commun..

[8] Welter M-L (2021). Deep brain stimulation of the subthalamic, accumbens, or caudate nuclei for patients with severe obsessive-compulsive disorder: a randomized crossover controlled study. Biol. Psychiatry.

[9] Saxena S, Brody AL, Schwartz JM, Baxter LR (1998). Neuroimaging and frontal-subcortical circuitry in obsessive-compulsive disorder. Br. J. Psychiatry Suppl..

[10] Baxter LR (1992). Caudate glucose metabolic rate changes with both drug and behavior therapy for obsessive-compulsive disorder. Arch. Gen. Psychiatry.

[11] Bijanki KR (2021). Defining functional brain networks underlying obsessive-compulsive disorder (OCD) using treatment-induced neuroimaging changes: a systematic review of the literature. J. Neurol. Neurosurg. Psychiatry.

[12] Gittis AH, Nelson AB, Thwin MT, Palop JJ, Kreitzer AC (2010). Distinct roles of GABAergic interneurons in the regulation of striatal output pathways. J. Neurosci..

[13] Mallet N, Moine CL, Charpier S, Gonon F (2005). Feedforward inhibition of projection neurons by fast-spiking GABA interneurons in the rat striatum in vivo. J. Neurosci..

[14] Planert H, Szydlowski SN, Hjorth JJJ, Grillner S, Silberberg G (2010). Dynamics of synaptic transmission between fast-spiking interneurons and striatal projection neurons of the direct and indirect pathways. J. Neurosci..

[15] Silberberg G, Bolam JP (2015). Local and afferent synaptic pathways in the striatal microcircuitry. Curr. Opin. Neurobiol..

[16] Lee K (2017). Parvalbumin interneurons modulate striatal output and enhance performance during associative learning. Neuron.

[17] O’Hare JK (2017). Striatal fast-spiking interneurons selectively modulate circuit output and are required for habitual behavior. eLife.

[18] Owen SF, Berke JD, Kreitzer AC (2018). Fast-spiking interneurons supply feedforward control of bursting, calcium, and plasticity for efficient learning. Cell.

[19] Lee, C. R. et al. Opposing influence of sensory and motor cortical input on striatal circuitry and choice behavior. *Curr. Biol.* **29**, 1313–1323.e5 (2019). 10.1016/j.cub.2019.03.028 PMC6482065 30982651

[20] Burguière E, Monteiro P, Mallet L, Feng G, Graybiel AM (2015). Striatal circuits, habits, and implications for obsessive-compulsive disorder. Curr. Opin. Neurobiol..

[21] Kataoka, Y. et al. Decreased number of parvalbumin and cholinergic interneurons in the striatum of individuals with Tourette syndrome. *J. Comp. Neurol.* **518**, 277–291 (2010). 10.1002/cne.22206 PMC2846837 19941350

[22] Kalanithi PSA (2005). Altered parvalbumin-positive neuron distribution in basal ganglia of individuals with Tourette syndrome. Proc. Natl Acad. Sci. USA.

[23] Briones, B. A. et al. Perineuronal nets in the dorsomedial striatum contribute to behavioral dysfunction in mouse models of excessive repetitive behavior. *Biol. Psychiatry Glob. Open Sci.* **2**, 460–469 (2021). 10.1016/j.bpsgos.2021.11.005 PMC9616293 36324654

[24] Welch JM (2007). Cortico-striatal synaptic defects and OCD-like behaviours in Sapap3-mutant mice. Nature.

[25] Ramírez-Armenta KI (2022). Optogenetic inhibition of indirect pathway neurons in the dorsomedial striatum reduces excessive grooming in Sapap3-knockout mice. Neuropsychopharmacology.

[26] Hadjas LC (2020). Projection-specific deficits in synaptic transmission in adult Sapap3-knockout mice. Neuropsychopharmacology.

[27] Pascoli V (2018). Stochastic synaptic plasticity underlying compulsion in a model of addiction. Nature.

[28] Oh SW (2014). A mesoscale connectome of the mouse brain. Nature.

[29] Foster NN (2021). The mouse cortico–basal ganglia–thalamic network. Nature.

[30] Hintiryan H (2016). The mouse cortico-striatal projectome. Nat. Neurosci..

[31] Rotge J-Y (2009). Meta-analysis of brain volume changes in obsessive-compulsive disorder. Biol. Psychiatry.

[32] Rotge J-Y (2010). Gray matter alterations in obsessive-compulsive disorder: an anatomic likelihood estimation meta-analysis. Neuropsychopharmacology.

[33] Simon D, Kaufmann C, Müsch K, Kischkel E, Kathmann N (2010). Fronto-striato-limbic hyperactivation in obsessive-compulsive disorder during individually tailored symptom provocation. Psychophysiology.

[34] Lei, H., Lai, J., Sun, X., Xu, Q. & Feng, G. Lateral orbitofrontal dysfunction in the Sapap3 knockout mouse model of obsessive–compulsive disorder. *J. Psychiatry Neurosci.* **44**, 120–131 (2019). 10.1503/jpn.180032 PMC6397042 30403026

[35] Manning, E. E., Geramita, M. A., Piantadosi, S. C., Pierson, J. L. & Ahmari, S. E. Distinct patterns of abnormal lateral orbitofrontal cortex activity during compulsive grooming and reversal learning normalize after fluoxetine. *Biol. Psychiatry* **93**, 989–999 (2023). 10.1016/j.biopsych.2021.11.018 35094880

[36] Owen SF, Liu MH, Kreitzer AC (2019). Thermal constraints on in vivo optogenetic manipulations. Nat. Neurosci..

[37] Senova S (2017). Experimental assessment of the safety and potential efficacy of high irradiance photostimulation of brain tissues. Sci. Rep..

[38] Gage GJ, Stoetzner CR, Wiltschko AB, Berke JD (2010). Selective activation of striatal fast-spiking interneurons during choice execution. Neuron.

[39] Martiros, N., Burgess, A. A. & Graybiel, A. M. Inversely active striatal projection neurons and interneurons selectively delimit useful behavioral sequences. *Curr. Biol.* **28**, 560–573.e5 (2018). 10.1016/j.cub.2018.01.031 PMC5820126 29429614

[40] Corbit VL, Manning EE, Gittis AH, Ahmari SE (2019). Strengthened inputs from secondary motor cortex to striatum in a mouse model of compulsive behavior. J. Neurosci..

[41] Dejean C (2016). Prefrontal neuronal assemblies temporally control fear behaviour. Nature.

[42] Karalis N (2016). 4-Hz oscillations synchronize prefrontal-amygdala circuits during fear behavior. Nat. Neurosci..

[43] Provenza NR (2021). Long-term ecological assessment of intracranial electrophysiology synchronized to behavioral markers in obsessive-compulsive disorder. Nat. Med..

[44] Nuttin B, Cosyns P, Demeulemeester H, Gybels J, Meyerson B (1999). Electrical stimulation in anterior limbs of internal capsules in patients with obsessive-compulsive disorder. Lancet.

[45] Mallet L (2008). Subthalamic nucleus stimulation in severe obsessive-compulsive disorder. N. Engl. J. Med..

[46] Li N (2021). A unified functional network target for deep brain stimulation in obsessive-compulsive disorder. Biol. Psychiatry.

[47] Tyagi H (2019). A randomized trial directly comparing ventral capsule and anteromedial subthalamic nucleus stimulation in obsessive-compulsive disorder: clinical and imaging evidence for dissociable effects. Biol. Psychiatry.

[48] Li N (2020). A unified connectomic target for deep brain stimulation in obsessive-compulsive disorder. Nat. Commun..

[49] Graat I (2021). Long-term outcome of deep brain stimulation of the ventral part of the anterior limb of the internal capsule in a cohort of 50 patients with treatment-refractory obsessive-compulsive disorder. Biol. Psychiatry.

[50] Aouizerate B (2009). Distinct striatal targets in treating obsessive-compulsive disorder and major depression: case report. J. Neurosurg..

[51] Spix TA (2021). Population-specific neuromodulation prolongs therapeutic benefits of deep brain stimulation. Science.

[52] Zrenner, C., Belardinelli, P., Müller-Dahlhaus, F. & Ziemann, U. Closed-loop neuroscience and non-invasive brain stimulation: a tale of two loops. *Front. Cell. Neurosci.* **10**, 92 (2016). 10.3389/fncel.2016.00092 PMC4823269 27092055

[53] Ghasemi P, Sahraee T, Mohammadi A (2018). Closed- and open-loop deep brain stimulation: methods, challenges, current and future aspects. J. Biomed. Phys. Eng..

[54] van den Boom BJG (2023). Unraveling the mechanisms of deep-brain stimulation of the internal capsule in a mouse model. Nat. Commun..

[55] Paz JT (2013). Closed-loop optogenetic control of thalamus as a tool for interrupting seizures after cortical injury. Nat. Neurosci..

[56] Voigts J, Siegle JH, Pritchett DL, Moore CI (2013). The flexDrive: an ultra-light implant for optical control and highly parallel chronic recording of neuronal ensembles in freely moving mice. Front. Syst. Neurosci..

[57] Orito K, Chida Y, Fujisawa C, Arkwright PD, Matsuda H (2004). A new analytical system for quantification scratching behaviour in mice. Br. J. Dermatol..

[58] Lamothe H (2023). The Sapap3^−/−^ mouse reconsidered as a comorbid model expressing a spectrum of pathological repetitive behaviours. Transl Psychiatry.

[59] Mathis A (2018). DeepLabCut: markerless pose estimation of user-defined body parts with deep learning. Nat. Neurosci..

[60] Nath T (2019). Using DeepLabCut for 3D markerless pose estimation across species and behaviors. Nat. Protoc..

[61] Insafutdinov, E., Pishchulin, L., Andres, B., Andriluka, M. & Schiele, B. DeeperCut: a deeper, stronger, and faster multi-person pose estimation model. In *European Conference on Computer Vision* (eds. Leibe, B. et al.) 34–50 (Springer, 2016).

[62] He, K., Zhang, X., Ren, S. & Sun, J. *2016 IEEE Conference on Computer Vision and Pattern Recognition (CVPR)* (IEEE, 2016).

[63] Siegle JH (2017). Open Ephys: an open-source, plugin-based platform for multichannel electrophysiology. J. Neural Eng..

[64] Mitra, P. & Bokil, H. *Observed Brain Dynamics* (Oxford Univ. Press, 2008).

[65] Paxinos, G. & B. J. Franklin, K. *The Mouse Brain in Stereotaxic Coordinates* Vol. 1 (Academic Press, 2001).

[66] Hartig, F. DHARMa: residual diagnostics for hierarchical (multi-level/mixed) regression models. R package version v0.4.6. https://cran.r-project.org/web/packages/DHARMa/vignettes/DHARMa.html (2022).

[67] Dunn, P. K. & Smyth, G. K. Randomized quantile residuals. *J. Comput. Graph. Stat.* **5**, 236–244 (1996).

[68] Page EB (1963). Ordered hypotheses for multiple treatments: a significance test for linear ranks. J. Am. Stat. Assoc..

[69] Lenth, R., Singmann, H., Love, J., Buerkner, P., & Herve, M. Package “Emmeans”. R package version v.1.8.2. https://cran.r-project.org/web/packages/emmeans/index.html (2018).

