# Supplementary material for: Closed-loop recruitment of striatal interneurons prevents compulsive-like grooming behaviors
Source: Nat Neurosci. 2024 May 1;27(6):1148–56. doi: 10.1038/s41593-024-01633-3 (PMC11156588; doi:10.1038/s41593-024-01633-3)
Supplement: Supplementary file 1 — Supplementary Figs. 1–3 and Tables 1 and 2. [file 41593_2024_1633_MOESM1_ESM.pdf]

nature neuroscience

Article https://doi.org/10.1038/s41593-024-01633-3

Closed-loop recruitment of striatal interneurons prevents compulsive-like grooming behaviors

In the format provided by the authors and unedited

Supplementary Figure 1

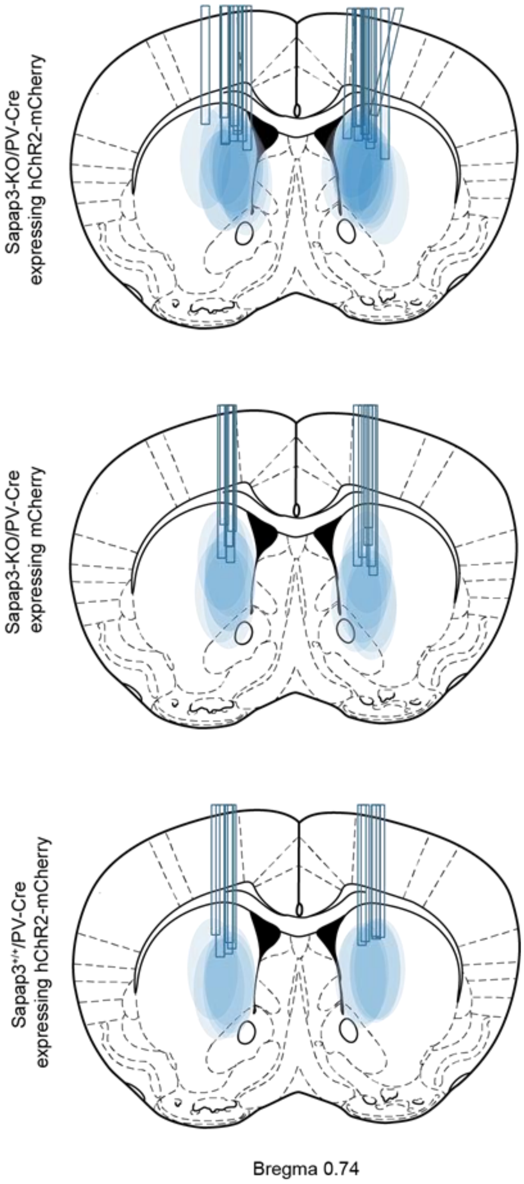

Reconstruction of optogenetically stimulated striatal area. Individual striatal fiber placements were confirmed *post-hoc* by comparing fiber tracks in histological coronal sections with measured fiber lengths of the detached implant after perfusion of the implanted animals. We simulated the optogenetically excited striatal area using an in vivo inspired Monte Carlo-based simulator to generate a predicted light propagation pattern[55] and irradiance of ~1 mW/mm^2 for a reliable opsin activation[56–58]. These reconstructions in three experimental cohorts of Sapap3-KO/PV-Cre mice expressing hChR2-mCherry, of Sapap3-KO/PV-Cre mice expressing mCherry, and of Sapap3$^{+/+}$/PVCre mice expressing hChR2-mCherry are summarized in separate panels from top to bottom, respectively.

Supplementary Figure 2.

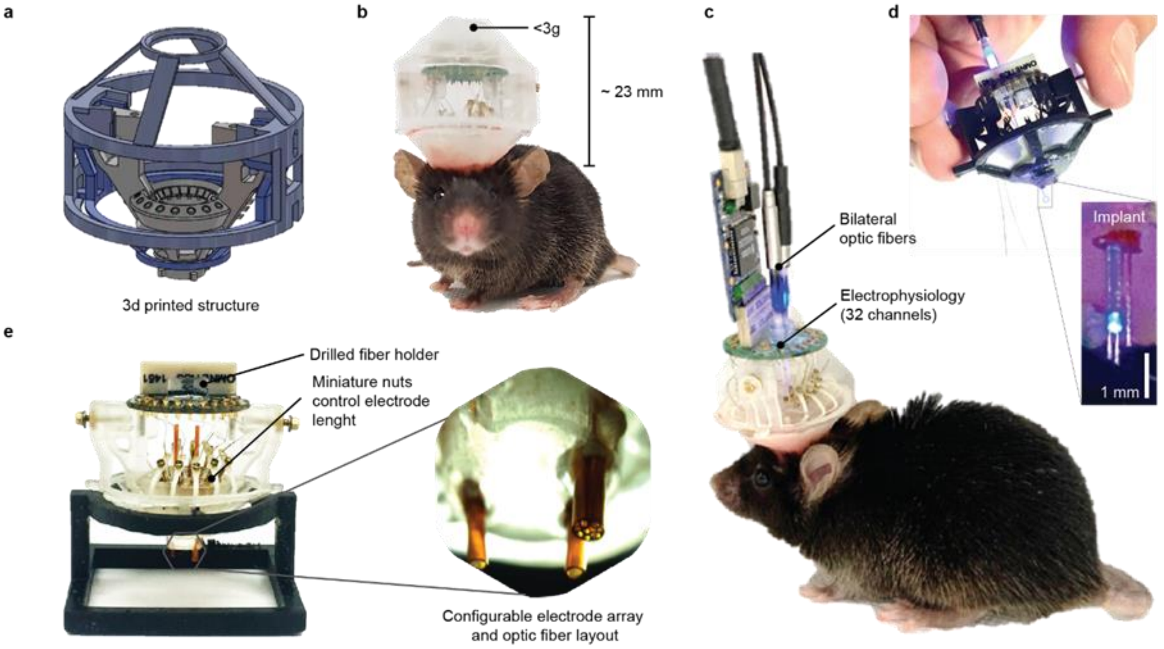

Cranial implant design for chronic electrophysiological recordings and optogenetic neuromodulation in freely moving mice. (**a**) Isometric view of the 3D model structure depicting the three individual printable pieces that assemble the implant: the lower protection cone, the removable protection cap and the element for adjusting tetrode positions ('drive'), which is a custom-adapted version of the "flexDrive" model[59]. (**b**) Picture of a mouse implanted with our custom-built chronic device, including the protection cap. (**c**) Picture of a chronically implanted mouse tethered for electrophysiology activity recording and optogenetic stimulation. (**d**) Picture of the implant during testing of optical power through optic fibers. Close-up view of an extracted implant after experimental procedures, showing one striatal fiber and surrounding tetrodes for optotagging of recorded PVIs. (**e**) Picture of the custom-adapted configurable 3D printed driving mechanism targeting multiple brain regions and allowing for the adjustment of individual tetrode depths for optimising chronic electrophysiological recordings over the duration of several weeks. Close-up bottom view of the implant showing the bottom end of the tubing inside of which either optical fibers or up to eight tetrodes can be placed.

Supplementary Figure 3.

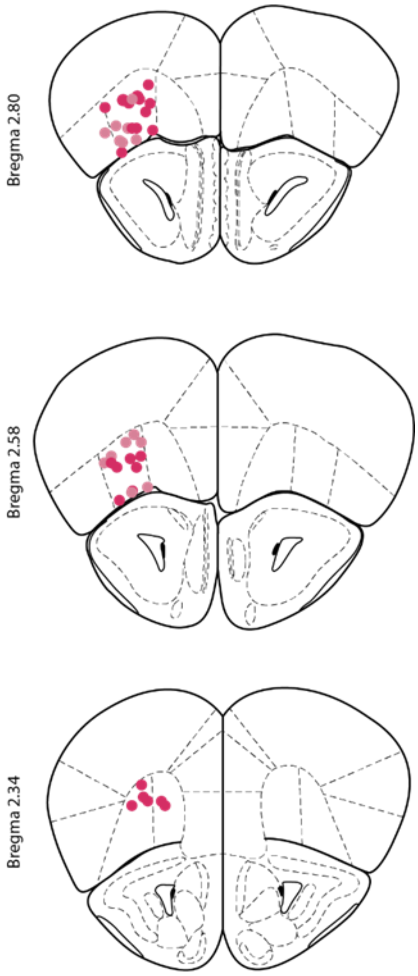

Post-hoc **histological confirmation of tetrode tip placements in the lOFC.** Reconstruction of individual tetrode tips as electrophysiological recording sites in the lOFC, mapped to coronal sections of a mouse brain atlas[66]. The location of each tetrode tip was determined based on electrolytic mark lesions in prefrontal coronal sections or measuring of tetrode lengths of the detached implant post perfusion. All illustrated recording sites were used for the LFP signature (n = 10 Sapap3-KO/PV-Cre mice). Recording sites, which were only used for LFP signature characterization (light magenta), are graphically distinguished from recoding sites, which in addition to LFP signature characterization were used for closed-loop stimulation experiments (dark magenta; n = 5 Sapap3-KO/PV-Cre mice).

Supplementary Table 1

Test result	Description	Consequence in a closed-loop experiment
True positive (TP)	A grooming is correctly predicted.	Targeted optogenetic stimulation and analysis pause for 4s.
True negative (TN)	A behaviour other than grooming is predicted, and the outcome is indeed other behaviour.	No stimulation. These trials represent correct stimulator holdback.
False positive (FP)	A grooming is predicted by the algorithm, but the outcome is not a grooming bout.	Unnecessary optogenetic stimulation.
False negative (FN)	A behaviour other than grooming is predicted, and the outcome is a grooming bout.	No stimulation. These trials represent missed grooming bouts.

Classification outcome insights. Overview detailing True Positives, True Negatives, False Positives, and False Negatives, including their definitions and impact on the experiment's results.

Supplementary Table 2

Metric	Definition
Sensitivity	$\frac{TP}{TP + FN}$
Precision	$\frac{TP}{TP + FP}$
Accuracy	$\frac{TP + TN}{TP + TN + FP + FN}$
Specificity	$\frac{TN}{TN + FP}$

Classification metrics formulae. Key performance indicators—sensitivity, precision, accuracy, and specificity—used to assess and interpret the effectiveness of a classification model.
