# Supplementary material for: Closed-loop recruitment of striatal interneurons prevents compulsive-like grooming behaviors
Source: Nat Neurosci. 2024 May 1;27(6):1148–56. doi: 10.1038/s41593-024-01633-3 (PMC11156588; doi:10.1038/s41593-024-01633-3)
Supplement: Supplementary file 2 — Reporting Summary [file 41593_2024_1633_MOESM2_ESM.pdf]

nature portfolio

Corresponding author(s): Eric Burguière

Last updated by author(s): 15/03/2024

Reporting Summary

Nature Portfolio wishes to improve the reproducibility of the work that we publish. This form provides structure for consistency and transparency in reporting. For further information on Nature Portfolio policies, see our Editorial Policies and the Editorial Policy Checklist.

Statistics

For all statistical analyses, confirm that the following items are present in the figure legend, table legend, main text, or Methods section.

n/a	Confirmed	
☐	☒	The exact sample size (*n*) for each experimental group/condition, given as a discrete number and unit of measurement
☐	☒	A statement on whether measurements were taken from distinct samples or whether the same sample was measured repeatedly
☐	☒	The statistical test(s) used AND whether they are one- or two-sided<br>*Only common tests should be described solely by name; describe more complex techniques in the Methods section.*
☐	☒	A description of all covariates tested
☐	☒	A description of any assumptions or corrections, such as tests of normality and adjustment for multiple comparisons
☐	☒	A full description of the statistical parameters including central tendency (e.g. means) or other basic estimates (e.g. regression coefficient) AND variation (e.g. standard deviation) or associated estimates of uncertainty (e.g. confidence intervals)
☐	☒	For null hypothesis testing, the test statistic (e.g. *F*, *t*, *r*) with confidence intervals, effect sizes, degrees of freedom and *P* value noted<br>*Give P values as exact values whenever suitable.*
☒	☐	For Bayesian analysis, information on the choice of priors and Markov chain Monte Carlo settings
☒	☐	For hierarchical and complex designs, identification of the appropriate level for tests and full reporting of outcomes
☒	☐	Estimates of effect sizes (e.g. Cohen's *d*, Pearson's *r*), indicating how they were calculated

Our web collection on statistics for biologists contains articles on many of the points above.

Software and code

Policy information about availability of computer code

Data collection	(1) Extracellular recordings were either acquired using the commercial Intan hardware acquisition system (RHD2000 USB Interface Board with the RHD 32-Channel Headstage, Intan Technologies, CA, USA) and the commercial software by Intan (RHD USB Interface Board software, Version 1.5.4,) or the open-source software Open Ephys G.U.I. (v0.5.5.3).<br>(2) For the closed-loop experiments, we used the open-source library RHD Matlab Toolbox for Windows (Version 1.2.2)<br>(3) Histology: striatal sections were imaged using a slide scanner (Axio Scan.Z1, Zeiss) with its commercial software ZEN. (ZEN. Blue 3.1, ZEISS).

Data analysis	1. Local field potential analysis: custom Matlab routines that use the Chronux Matlab package (http://chronux.org) and the Matlab Signal Processing toolbox.<br>2. Spike analysis: Spike sorting was performed offline using the valley-seeking prevalent method in Offline sorter (Version 3.3.5, Plexon Inc.) and NeuroExplorer (Version 4, Nex Technologies, Colorado, U.S.A.)<br>3. Statistical analysis: All statistical analyses were performed using either Prism (GraphPad Software Inc, version 8.0.1) or R (R Development Core Team, 2021, version 4.1.1) with the following packages: DHARMa (v0.4.6), glmmTMB (v1.1.4), car (v3.1-0), DescTools (v0.99.46, for PageTest), emmeans (v1.8.2).<br>4. Histology: Striatal sections were analyzed and mapped using ZEN software (ZEN. Blue 2.3, ZEISS)<br>5. Closed-loop experiments used the commercial Matlab R2019b Signal Processing toolbox and custom algorithms described in Figure 3 of the present manuscript.<br>6. Body parts tracking and behaviour annotation: For tracking body parts in videos we used the open-source Python toolkit DeepLabCut (version 2.2.1, with CUDA Toolkit 11.0 and Tensorflow 2.3.0) and Matlab custom scripts (Matlab 2022b). For video scoring we used the software Kinovea (version 0.8.15)<br>All custom MATLAB code generated in this work is openly available in the repository: https://github.com/LizbethMG/2024_Mondragon-Gonzalez_NatureNeuroscience

For manuscripts utilizing custom algorithms or software that are central to the research but not yet described in published literature, software must be made available to editors and reviewers. We strongly encourage code deposition in a community repository (e.g. GitHub). See the Nature Portfolio guidelines for submitting code & software for further information.

Data

Policy information about availability of data

All manuscripts must include a data availability statement. This statement should provide the following information, where applicable:

- Accession codes, unique identifiers, or web links for publicly available datasets
- A description of any restrictions on data availability
- For clinical datasets or third party data, please ensure that the statement adheres to our policy

The data supporting this study's findings are available in the publicly accessible repository: (DOI 10.17605/OSF.IO/KDMJT).
Source data are provided with this paper.

Field-specific reporting

Please select the one below that is the best fit for your research. If you are not sure, read the appropriate sections before making your selection.

☒ Life sciences ☐ Behavioural & social sciences ☐ Ecological, evolutionary & environmental sciences

For a reference copy of the document with all sections, see nature.com/documents/nr-reporting-summary-flat.pdf

Life sciences study design

All studies must disclose on these points even when the disclosure is negative.

Sample size	The sample size for all experiments was determined based on the variance observed in previous similar experiments and practical considerations (DOI: 10.1038/nn.3269, DOI: 10.1038/s41386-021-01161-9, DOI: 10.1038/s41467-023-41026-x). Specifically, the sample size for animal experiments was as follows:<br>On/Off optogenetic stimulation experiment: N=10, Sapap3-KO/PVCre mice expressing hChR2; N=6 Sapap3-KO/PVCre mice injected with a control virus (i.e. only expressing the fluorophore marker mCherry) with N=3 putative PV interneurons for opto-tagging demonstration. N=5 wildtypes mice expressing hChR2.<br>LFP analysis: N=10 Sapap3-KO/PVCre expressing either hChR2 or mCherry only.<br>Algorithm validation: N = 7 Sapap3-KO/PVCre mice injected with hChR2; the validation was performed during phases without light stimulation.<br>Closed-loop experiment: N=5 Sapap3-KO/PVCre mice injected with hChR2.
Data exclusions	Six out of 45 tetrode recording sites that were detected slightly outside of the lOFC were excluded from LFP analyses.
Replication	All experiments were successfully replicated as follows:<br>1. All optogenetic ON-OFF experiments were replicated in intra-sessions (5 repetitions per condition and animal) and inter-sessions (3 repetitions per animal).<br>2. All optogenetic Closed-loop experiments were replicated in intra-sessions (5 repetitions per condition and per animal) and inter-sessions (3 repetitions per animal).<br>3. All grooming related LFP recordings were replicated (mean 25 observations per animal with ten animals).<br>4. All experiments to test the algorithm performance were replicated in N = 7 animals (10 minutes per animal).
Randomization	Animals were chosen based on having the appropriate range of age and weight at the moment of the experiment. Given the heterogeneity of the phenotype expression (Manning et al., Sci Reports, 2021), we very briefly screened randomly in the cages for Sapap3-KO mice with overexpression of the self-grooming phenotype but without skin lesions. The order of testing ON/OFF optogenetic stimulation in Sapap3-KO/PVCre mice expressing either hChR2 or control virus expressing only mCherry, or wildtypes mice expressing hChR2 was randomized. Further randomization was not relevant to this study as each animal served as its control in ON/OFF stimulation conditions and the Closed-Loop stimulation paradigm in ON/OFF/Yoked conditions (5 replications within one session, three sessions on different days; see "Replication"

paragraph). For the LFP study, randomization was also not required as each animal again served as its own control, i.e. each per-grooming LFP was compared to its baseline activity.

Blinding

For data collection, the selection of animals was based solely on matched age and weight ranges and the expression of the overgrooming phenotype in Sapap3-KO mice. The group allocation was then decided according to animal genotype and/or the treatment previously administered to the animal, i.e. the type of virus injected.
All data analyses were conducted either offline (manual behavioural quantification with blind scoring of events, including grooming, scratching, and walking, across all experiments) or were fully automated, not requiring group assignment or susceptible to human bias. These include LFP analysis, closed-loop code routines, and the global activity analysis, as detailed in the Behavioural Assessment section; thus, blinding was not necessary. For manual behavioural quantification, we implemented inter-rater control to validate the manual annotation of video frames. Three naïve raters, trained by an expert to recognize grooming using an instructional video, were assigned to annotate five recording sessions. Each 36-minute video represented a Sapap3-KO mouse in a single session from the closed-loop experiments, consisting of 60 blocks of 3 minutes each, for a total of 90 grooming events.

Reporting for specific materials, systems and methods

We require information from authors about some types of materials, experimental systems and methods used in many studies. Here, indicate whether each material, system or method listed is relevant to your study. If you are not sure if a list item applies to your research, read the appropriate section before selecting a response.

Materials & experimental systems

n/a	Involved in the study	
☐	☒	Antibodies
☒	☐	Eukaryotic cell lines
☒	☐	Palaeontology and archaeology
☐	☒	Animals and other organisms
☒	☐	Human research participants
☒	☐	Clinical data
☒	☐	Dual use research of concern

Methods

n/a	Involved in the study	
☒	☐	ChIP-seq
☒	☐	Flow cytometry
☒	☐	MRI-based neuroimaging

Antibodies

Antibodies used

We used the following primary and secondary antibodies and dilutions:
1. anti-red fluorescent protein antibody (anti-RFP; polyclonal rabbit, Rockland, #600-401-379, Lot #35634; dilution: 1:1000)
2. anti-parvalbumin antibody (anti-PV GP 72; polyclonal guinea pig, Swant, dilution 1:5000)
3. goat-anti-rabbit Cy3-conjugated antibody (AffiniPure, polyclonal, Lot 106489; dilution 1:400)
4. goat-anti-guinea pig Alexa488 antibody (Invitrogen, polyclonal, Lot #145863, dilution: 1:400).

Validation

Antibodies were validated in coronal brain sections (section thickness 40µm as all coronal samples of the presented study) of a mouse, expressing virally transfected mCherry in the dorsal striatum. Specificity of fluorescent labelling was achieved through verification of expected expression patterns as well as negative control sections, which were incubated only in secondary but not in primary antibody blocking solution. Optimal dilutions yielding bright specific signal and low background fluorescence were determined from a series of dilution tests (Rockland anti-RFP #600-401-379: dilutions of 1:1000, 1:1000; Swant anti-PV GP 72: dilutions of 1:3000, 1:5000, 1:10.000; all tested dilutions fell into the range corresponding to manufacturer's recommendations for histological immunofluorescence labelling).

Animals and other organisms

Policy information about studies involving animals; ARRIVE guidelines recommended for reporting animal research

Laboratory animals

Mice were maintained under a consistent 12-hour light cycle (from 6:00 a.m. to 6:00 p.m.) and a 12-hour dark cycle (from 6:00 p.m. to 6:00 a.m.), in a temperature-controlled environment ranging from 20°C to 24°C and humidity levels between 40% and 60%. They had unlimited access to standard chow and water.
Breeding:
-The Sapap3-knockout (Sapap3-/-) mouse line on C57BL6/J background (B6.129-Dlgap3tm1Gfng/J ; Jackson Laboratory stock #008733) was provided by Drs. G. Feng and A.M. Graybiel (Massachusetts Institute of Technology, Cambridge, U.S.A.) and backcrossed on C57BL/6 background strain (Jackson Laboratory) every 5-10 generations. Heterozygous male and female Sapap3+/- mice from this breeding were used at the age of 3-4 months to be crossed with mice from the (Pvalb)-Cre line.
-Sapap3+/− were crossed with parvalbumin (PV) (Pvalb)-Cre line (B6;129P2-Pvalbtm1(cre)Arbr/J; Jackson Laboratory stock #008069; males and females, 3-4 months old; provided by Dr A. Bacci, Paris Brain Institute, France) to obtain heterozygous Cre-positive Sapap3 +/+ and Sapap3-/- mice.
Experimental studies:
We used 17 male Sapap3-/- :: PV-Cre/wt (average initial age = 8.6 ±1.4 months, average weight = 30.5 ±3.7 g) and 5 age-matched Sapap3+/+::PV-Cre/wt (average initial age = 9.1 ±1 months, average weight = 39.3 ±4 g).

Wild animals

The study did not involve wild animals.

Field-collected samples	The study did not involve samples collected from the field.
Ethics oversight	The French Ministry of Higher Education, Research and Innovation (APAFIS #1418-2015120217347265 and #31141-2021042017105235) has approved all experimental procedures.

Note that full information on the approval of the study protocol must also be provided in the manuscript.
